# Lactylation‐Driven NUPR1 Promotes Immunosuppression of Tumor‐Infiltrating Macrophages in Hepatocellular Carcinoma

**DOI:** 10.1002/advs.202413095

**Published:** 2025-04-30

**Authors:** Jialiang Cai, Peiling Zhang, Yufan Cai, Guiqi Zhu, Shiping Chen, Lina Song, Junxian Du, Biao Wang, Weixing Dai, Jian Zhou, Jia Fan, Yiyi Yu, Zhi Dai

**Affiliations:** ^1^ Liver Cancer Institute Zhongshan Hospital Fudan University Shanghai 200032 China; ^2^ State Key Laboratory of Genetic Engineering Fudan University Shanghai 200032 China; ^3^ Key Laboratory of Carcinogenesis and Cancer Invasion Fudan University Ministry of Education Shanghai 200032 China; ^4^ Department of General Surgery Zhongshan Hospital Fudan University Shanghai 200032 China; ^5^ Department of Liver Surgery and Transplantation Zhongshan Hospital Fudan University Shanghai 200032 China; ^6^ Research Unit of Liver Cancer Recurrence and Metastasis Chinese Academy of Medical Sciences Beijing 100000 China; ^7^ Department of Radiation Oncology Zhongshan Hospital Fudan University Shanghai 200032 China; ^8^ Department of Colorectal Surgery Fudan University Shanghai Cancer Center Shanghai 200032 China; ^9^ Department of Oncology Shanghai Medical College Fudan University Shanghai 200032 China; ^10^ Department of Medical Oncology Zhongshan Hospital Fudan University 180 Fenglin Road Shanghai 200032 China; ^11^ Cancer Center Zhongshan Hospital Fudan University Shanghai 200032 China

**Keywords:** histone lactylation, immune checkpoint blockade, MAPK pathway, nuclear protein 1, tumor‐associated macrophages (TMA)

## Abstract

While checkpoint immunotherapy effectively mobilizes T‐cell responses against tumors, its success in hepatocellular carcinoma (HCC) is frequently undermined by immunosuppressive myeloid cells within the tumor microenvironment. This study investigates the role of nuclear protein 1 (NUPR1), a gene prominently expressed in tumor‐associated macrophages (TAMs), in mediating this suppression and influencing immunotherapy outcomes. Through comprehensive analysis of single‐cell RNA sequencing (scRNA‐seq) datasets and functional assays in vitro and in vivo, NUPR1 is identified as a critical regulator within TAMs. The upregulation of NUPR1 is associated with enhanced M2 macrophage polarization and increased expression of immune checkpoints PD‐L1 and SIRPA, resulting in CD8+ T cell exhaustion and a diminished response to immunotherapy. Mechanistically, NUPR1 inhibits the ERK and JNK signaling pathways, thereby creating an immunosuppressive milieu conducive to tumor progression. Additionally, tumor‐derived lactate is shown to upregulate NUPR1 expression in macrophages via histone lactylation, perpetuating a feedback loop that intensifies immune suppression. Pharmacological targeting of NUPR1 reverses M2 polarization, curtails tumor growth, and augments the efficacy of PD‐1 blockade in preclinical models, positioning NUPR1 as both a potential biomarker for immunotherapy responsiveness and a therapeutic target to boost immunotherapy efficacy in HCC.

## Graphical abstract

Cai et al. report that tumor‐derived lactate induces NUPR1 expression via H3K18 lactylation, which can promote the pro‐tumor M2 macrophage polarization through ERK and JNK signaling pathways. Treatment with NUPR1 selective inhibitor sensitizes synergizes with anti‐PD‐1 therapy in murine preclinical models.

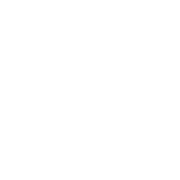

## 1 Introduction

Hepatocellular carcinoma (HCC) ranks among the deadliest cancers globally, presenting a substantial health burden due to its high incidence.^[^ ^1^, ^2^ ^]^ Traditionally, hepatectomy has been the cornerstone of treatment; however, its effectiveness is limited by high recurrence rates post‐surgery.^[^ ^3^ ^]^ Recently, immunotherapy, especially targeting the programmed cell death (/ligand) 1 (PD‐1/PD‐L1) axis, has emerged as pivotal.^[^ ^4^, ^5^, ^6^ ^]^ Despite its success in cancers like melanoma, response rates in HCC remain modest (15‐30%), and the overall survival benefits are often minimal.^[^ ^7^ ^]^ A key factor in this limited efficacy is the immunosuppressive tumor microenvironment (TME), which significantly contributes to resistance to immunotherapy in HCC.^[^ ^8^, ^9^ ^]^ Therefore, unraveling and targeting the mechanisms underpinning this immunosuppression is crucial to improve immunotherapy outcomes in patients with HCC.

The liver, as an immune‐privileged organ, contains a substantial proportion of macrophages, including tissue‐resident Kupffer cells and monocyte‐derived macrophages.^[^ ^10^ ^]^ These cells are central to the progression of HCC, serving dual roles: they can exert anti‐tumor effects through antigen presentation and phagocytosis^[^ ^11^ ^]^ or, conversely, promote tumor progression when reprogrammed into tumor‐associated macrophages (TAMs), akin to M2 macrophages.^[^ ^12^ ^]^ The remarkable plasticity of TAMs, characterized by their diverse phenotypic, metabolic, and functional profiles, not only underscores their role in immune suppression but also provides a window for therapeutic intervention.^[^ ^13^ ^]^ Deciphering the molecular signatures of TAMs and identifying key macrophage subsets are pivotal for reversing the TME's immunosuppressive state and enhancing the efficacy of immune checkpoint blockade (ICB) therapy.

NUPR1, or Nuclear Protein 1, is a transcriptional regulator involved in various cellular processes, including apoptosis, cell cycle regulation, and DNA repair,^[^ ^14^ ^]^ and is often overexpressed in several cancers such as pancreatic,^[^ ^15^ ^]^ breast,^[^ ^16^ ^]^ and liver cancers,^[^ ^17^, ^18^ ^]^ where it contributes to tumor survival, proliferation, and resistance to chemotherapy. Notably, compounds like Trifluoperazine and ZZW‐115, traditionally used for psychiatric symptoms, have been identified as selective inhibitors of NUPR1, highlighting its potential as a cancer therapy target.^[^ ^19^ ^]^ Nevertheless, the research has predominantly focused on NUPR1 within tumor cells, with its role in the TME remaining underexplored. Thus, a comprehensive analysis of how NUPR1 influences the TME is crucial for translating these findings into clinical applications. This could potentially enhance therapeutic strategies and provide a deeper understanding of tumor biology in relation to immune regulation.

This study leverages high‐throughput single‐cell RNA sequencing (scRNA‐seq) to reveal that NUPR1 is predominantly expressed in TAMs within the HCC microenvironment. Macrophages with high NUPR1 expression showed reduced pro‐inflammatory and T‐cell supportive functions compared to their NUPR1‐low counterparts. Pharmacological inhibition of NUPR1 sensitized HCC tumors to anti‐PD‐1 therapy in vivo. Additionally, we discovered that lactate from tumor cells induces histone H3K18 lactylation in macrophages, which upregulates NUPR1 transcription. This research unveils a novel role for NUPR1 in augmenting the efficacy of ICB therapy in HCC, providing insights into the molecular mechanisms of tumor immune evasion and outlining new strategies for targeting TAMs to boost T‐cell antitumor responses.

## 2 Results

### 2.1 NUPR1 is Highly Expressed in Tumor‐Associated Macrophages and Correlates with Poor Prognosis in Patients with HCC

**Figure 1:**
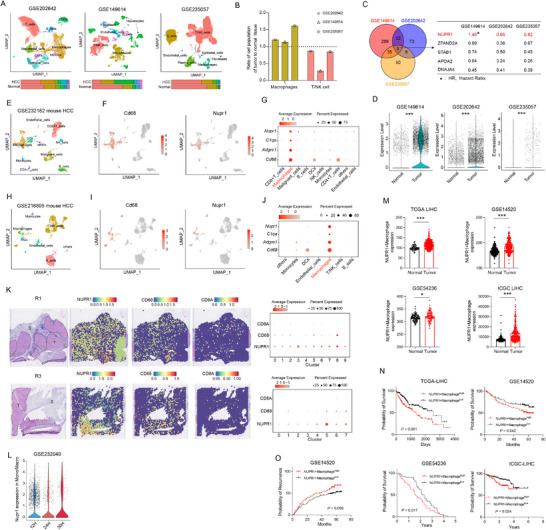
NUPR1 is highly expressed in macrophages and associated with prognosis in HCC. A) UMAP plots illustrating the scRNA‐seq data from three different datasets, and the bar graphs accompanying each UMAP plot represent the proportions of these cell populations in tumor versus normal tissues. B) Bar plots displaying the ratio of macrophage and T/NK cell populations in tumor tissues compared to normal tissues. C) The left side of the panel shows Venn diagrams for three datasets, each illustrating the differentially expressed genes in macrophages between tumor and normal tissues. The right side highlights the intersection of these datasets, identifying five common genes. D) Violin plots showing the expression of NUPR1 in macrophages from tumor tissues and normal tissues. E) UMAP plots depicting various cell types in murine HCC tumors from the GSE232182 dataset. F) UMAP plots illustrating the expression of *Cd68* and *Nupr1* across various cell types in the GSE232182 dataset. G) Dot plots displaying the expression of *Nupr1*, *C1qa*, *Adgre1*, and *Cd68* among different cell types in the GSE232182 dataset. H) UMAP plots depicting various cell types in murine HCC tumors from the GSE216805 dataset. I) UMAP plots illustrating the expression of *Cd68* and *Nupr1* across various cell types in the GSE216805 dataset. J) Dot plots displaying the expression of *Nupr1*, *C1qa*, *Adgre1*, and *Cd68* among different cell types in the GSE216805 dataset. K) H‐E staining (left) reveals the tissue morphology of spatial transcriptomics samples. The Spatial Violin Plot (middle) shows the expression of NUPR1, CD68, and CD8 across different spatial regions. The Dot Plot (right) illustrates the expression of NUPR1, CD68, and CD8 across various clusters from the GSE238264 dataset. T, tumor; S, stromal. L) Violin plots illustrating the expression levels of NUPR1 in macrophages migrating to tumors across different time periods. M) Bar graph displaying the expression differences of NUPR1+ macrophages between cancerous and normal tissues. N) Kaplan–Meier survival curves demonstrating that patients with HCC exhibiting high levels of NUPR1+ macrophages have poorer overall prognosis compared to those with lower expression levels. O) Kaplan–Meier survival curves demonstrating that patients with HCC exhibiting high expression of NUPR1+ macrophages have higher recurrence rates compared to those with lower expression. *n*s, not significant; * *P* < 0.05, ** *P* < 0.01, *** *P* < 0.001.

To explore alterations within the TME of hepatocellular carcinoma (HCC), we analyzed three publicly available single‐cell RNA sequencing (scRNA‐seq) datasets comprising both adjacent normal liver tissues and HCC tissues (**Figure** **1**A; Figure S1A, Supporting Information). Our analysis revealed a heightened presence of macrophages within tumors, alongside a reduction in T cells and NK cells (Figure 1B; Figure S1B, Supporting Information). Given the critical role of macrophages in the liver's immune landscape and their association with HCC progression, we further examined differential gene expression between macrophages in tumor and non‐tumor tissues. This analysis identified five common genes, including NUPR1, which has not been extensively studied in macrophages (Figure 1C,D; Figure S1C, Supporting Information). Violin plot analysis revealed that NUPR1 expression was markedly elevated in macrophages compared to other cell types, with this trend being particularly pronounced in the GSE149614 dataset (Figure S1D, Supporting Information). Subsequent analysis using two murine HCC scRNA‐seq datasets revealed that NUPR1 was predominantly expressed in macrophages, confirming the widespread high expression of NUPR1 in macrophages across species (Figure 1E–J; Figure S1D,E, Supporting Information). Subsequently, analysis of spatial transcriptome (ST) datasets of HCC revealed the co‐localization of NUPR1 with CD68 across multiple samples (Figure 1K; Figure S2, Supporting Information). Notably, NUPR1 expression was higher in the tumor regions compared to non‐tumor areas (Figure 1K), indicating its predominant expression in TAMs. Additionally, in a study by Liu et al.,^[^ ^20^ ^]^ which employed single‐cell expression profiling to monitor the temporal dynamics of circulating immune cells as they infiltrated the TME, our analysis revealed an increase in NUPR1 expression in intratumoral macrophages over time (Figure 1L). These data suggest that NUPR1 may play a crucial role in the formation of TAMs.

Following the integration of macrophage populations from three scRNA‐seq datasets using the Harmony algorithm (Figure S3A, Supporting Information), stratified macrophages into NUPR1‐high and NUPR1‐low subpopulations based on their mean NUPR1 expression (Figure S3B, Supporting Information). We then derived gene signatures for NUPR1+macrophage by identifying differential expressed genes between NUPR1‐high and NUPR1‐low subpopulations, while excluding genes not specific to monocytes/macrophages, as detailed in the Experimental Section. Analysis of RNA‐sequencing (RNA‐seq) data from The cancer genome atlas (TCGA), gene expression omnibus (GEO) databases, and international cancer genome consortium (ICGC) revealed that the NUPR1+ macrophage signature was significantly enriched in tumor tissues compared to adjacent normal tissues (Figure 1M). Additionally, patients exhibiting higher expression of the NUPR1+ macrophages were associated with significantly poorer overall survival (OS) (Figure 1N). Notably, stratifying patients into two groups based on median NUPR1 expression did not reveal a significant difference in OS between those with high and low NUPR1 expression (Figure S3C, Supporting Information). The data also revealed that patients with HCC had a significantly elevated recurrence rate in NUPR1‐high macrophages (Figure 1O). In contrast, stratification based on median NUPR1 expression did not show a significant difference in recurrence rate (Figure S3D, Supporting Information). This finding highlight that it is specifically the expression of NUPR1 within macrophages, instead of in other cell types, that drives the poor prognosis in patients with HCC. Subsequently, univariate and multivariate Cox regression analyses showed that higher expression of NNUPR1+macrophages was an independent predictor for postoperative OS (Table S1, Supporting Information). NUPR1 expression was examined across various cancer types, revealing the NUPR1+ macrophages were more abundant in tumors than in normal tissues (Figure S3E, Supporting Information). Individuals with elevated expression of the NUPR1+ macrophages exhibited poorer OS (Figure S3F, Supporting Information). Stratifying patients based on median NUPR1 expression again showed no significant difference in OS between NUPR1‐high and NUPR1‐low groups (Figure S3G, Supporting Information), underscoring the effect of NUPR1 expression specifically in macrophages on the unfavorable prognosis of patients across tumor types.

### 2.2 NUPR1+Macrophages Mediate the Induction of Immunosuppressive Microenvironment

**Figure 2:**
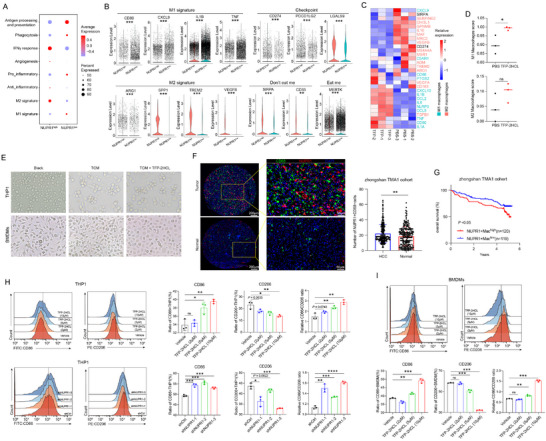
NUPR1 preserves the immunosuppressive phenotype in macrophages. A) Bubble plot showing the expression of various biological characteristics associated with macrophages in NUPR1low and NUPR1high macrophages. B) Violin plots illustrate the differences in macrophage characteristic genes between NUPR1low and NUPR1high macrophages. C) Heatmap showing the differential expression of genes related to M1 and M2 macrophages after treatment with NUPR1‐selective inhibitor TFP‐2HCL (10µM) in THP‐1 cells. D) Bar plots illustrating the differences in M1 and M2 macrophage signature scores between TFP‐treated THP1 cells and control cells. E) Images showing the morphology of control macrophages, TCM‐treated macrophages, and TCM plus TFP‐2HCL‐treated macrophages. F) Representative images and statistical analysis of mIF staining for CD68 and NUPR1 of HCC tissue (*n* = 239) and normal tissues (*n* = 225). G) Kaplan–Meier curves for OS of patients with HCC. H) Flow cytometric analysis of CD86 and CD206 in THP1 cells treated with TFP‐2HCL (2, 5, and 10 µm) or vehicle or transfected with shNUPR1 plasmid or control (*n* = 3). I) Flow cytometric analysis of CD86 and CD206 in BMDMs treated with TFP‐2HCL (2, 5, and 10 µm) or vehicle (*n* = 3). Results are representative of at least three independent experiments. Data are presented as mean ± SD. *n*s, not significant; * *P* < 0.05, ** *P* < 0.01, *** *P* < 0.001.

To investigate the role of NUPR1 in macrophages, we first extracted macrophage populations from three scRNA‐seq datasets. These macrophages were then integrated using the Harmony algorithm to ensure a consistent representation across datasets. Following integration, we computed the average expression level of NUPR1 across all macrophages. Macrophages exhibiting NUPR1 expression levels below the average were classified as NUPR1‐low, while those with expression levels above the average were categorized as NUPR1‐high. Gene Set Enrichment Analysis (GSEA) was then conducted on differentially expressed genes (DEGs) between NUPR1‐high and ‐low macrophages to identify enriched biological pathways. Our analysis demonstrated that pathways associated with immune suppression, such as the regulation of response to wounding, macrophage migration, and positive regulation of lipid transport, were enriched in the NUPR1‐high macrophages (Figure S4A, Supporting Information). Conversely, immune activation pathways, including positive regulation of cytokine production and alpha–beta T cell activation, were predominant in NUPR1‐low macrophages (Figure S4A, Supporting Information). Further analysis showed that NUPR1‐high macrophages exhibited increased signature scores for IFN‐γ response, angiogenesis, anti‐inflammation, and M2 macrophage characteristics, while NUPR1‐low macrophages demonstrated higher scores for antigen processing and presentation, phagocytosis, pro‐inflammatory responses, and M1 macrophage features (**Figure** **2**A). Specifically, genes associated with M1 macrophages, such as CD80 and Interleukin 1 Beta (IL1B), were upregulated in NUPR1‐low macrophages, whereas M2 macrophage‐related genes, including arginase 1 (ARG1), triggering receptor expressed on myeloid cells 2 (TREM2), and vascular endothelial growth factor B (VEGFB), were more highly expressed in NUPR1‐high macrophages (Figure 2B). Additionally, immune checkpoint CD274, programmed cell death 1 ligand 2 (PDCD1LG2), and galectin 9 (LGALS9) were prominently expressed in NUPR1‐high macrophages, along with the “don't eat me” signals signal regulatory protein alpha (SIRPA) and CD33 (Figure 2B). In contrast, the “eat me” checkpoint MER proto‐oncogene, tyrosine kinase (MERTK) was highly expressed in NUPR1‐low macrophages (Figure 2B). Furthermore, analysis of tumor cells in the GSE149614 dataset revealed that “don't eat me” ligands CD47, CD24, human leukocyte antigen A (HLA‐A), and HLA‐B were significantly upregulated in tumor cells from NUPR1‐high samples, indicating a potential effect of NUPR1+ macrophages on tumor cells (Figure S4B, Supporting Information). Utilizing data from the TCGA‐LIHC dataset, we observed a significant positive correlation between the NUPR1+ macrophage and the expression of immune checkpoints LGALS9, SIRPA, and CD274 (Figure S4C, Supporting Information). These findings underscore the complex role of NUPR1 in promoting immunosuppressive macrophages, warranting further investigation into NUPR1's influence on macrophage polarization. Multiplex immunofluorescence (mIF) was performed to validate our findings from public datasets. Our results showed that the number of CD68⁺CD206⁺NUPR1⁺ cells was significantly higher than that of CD68⁺CD86⁺NUPR1⁺ cells, indicating that NUPR1 is predominantly expressed in M2 macrophages (Figure S4D, Supporting Information). This experimental evidence further supports our conclusion that NUPR1 is associated with M2 macrophage polarization within the TME.

Neria et al. demonstrated that trifluoperazine dihydrochloride (TFP‐2HCL), an antipsychotic agent targeting NUPR1 in pancreatic cancer, binds to specific residues of the NUPR1 protein, inhibiting its function.^[^ ^21^ ^]^ Building on this, we applied TFP‐2HCL to inhibit NUPR1 and observed the effect on macrophages. RNA‐Seq was performed on THP1 cells treated with PBS or TFP‐2HCL, identifying 358 upregulated genes and 524 downregulated genes using a cutoff of Log2FC > 1 and *P* < 0.05 in TFP‐2HCL‐treated group (Figure S4E, Supporting Information). Heatmap analysis revealed that genes associated with M1 macrophages were upregulated, while genes associated with M2 macrophages were downregulated in TFP‐2HCL‐treated cells (Figure 2C). Additionally, the immune checkpoint SIRPA and CD274 were highly expressed in control cells (Figure 2C). Single‐sample gene set enrichment analysis (ssGSEA) analysis confirmed a significant increase in M1 macrophage signature scores following TFP‐2HCL treatment (Figure 2D). When THP‐1 cells and bone marrow‐derived macrophages (BMDMs) were incubated with tumor‐conditioned medium (TCM), the resulting TAMs displayed an elongated, spindle‐shaped morphology typical of M2 macrophages (Figure 2E). Interestingly, combining TCM with TFP‐2HCL induced a shift toward a more rounded morphology characteristic of M1 macrophages (Figure 2E). The TMA1 cohort of Zhongshan Hospital was used for mIF staining to examine the expression of NUPR1 and CD68 in tumor tissues compared to adjacent tissues. The findings revealed a significantly higher abundance of NUPR1+macrophages in tumor tissues (Figure 2F). Additionally, we observed that low expression of NUPR1+macrophages indicated a significantly better prognosis in patients with HCC than did the corresponding high expression, as evidenced by OS in the TMA1 cohort from Zhongshan Hospital (Figure 2G). Quantitative real‐time polymerase chain reaction (qRT‐PCR) analysis of TFP‐2HCL‐treated THP‐1 macrophages revealed increased expression of M1 macrophage markers nitric oxide synthase 2 (NOS2), CD86, tumor necrosis factor (TNF), and IL1B, alongside decreased expression of M2 macrophage markers CD206, ARG1, transforming growth factor beta 1 (TGFB1), and interleukin 10 (IL10) (Figure S4F, Supporting Information). To further investigate the effect of TFP‐2HCL on macrophage polarization, we performed PCR to measure the expression of NUPR1, PD‐L1, SIRPA, and macrophage polarization markers after treatment with different concentrations of TFP‐2HCL. The results showed that the expression levels of NUPR1, PD‐L1, and SIRPA decreased in a concentration‐dependent manner (Figure S4G, Supporting Information). In contrast, the expression levels of M2 macrophage markers decreased with increasing concentrations of TFP‐2HCL, while M1 macrophage markers showed a concentration‐dependent increase (Figure S4G, Supporting Information). These data indicate that TFP‐2HCL modulates macrophage polarization by affecting NUPR1 expression, further confirming the pivotal role of NUPR1 in macrophage polarization. Knockdown of NUPR1 in THP1 cells resulted in elevated NOS2 and CD86 expression while reducing CD206 and ARG1 expression (Figure S4H, Supporting Information). Flow cytometry analysis confirmed that TFP‐2HCL treatment or NUPR1 knockdown decreased CD206 expression while increasing CD86 expression in both THP‐1 cells and BMDMs (Figure 2H,I), highlighting NUPR1's role in M2 macrophage polarization, with its inhibition promoting M1 macrophage polarization. Western blotting revealed that NUPR1 knockdown or pharmacological inhibition led to a marked reduction in SIRPA and PD‐L1 expression (Figure S4I,J, Supporting Information), further confirming NUPR1's critical role in promoting the immunosuppressive characteristic of macrophages.

### 2.3 NUPR1 Promotes M2 Macrophages Polarization by Inhibiting the Mitogen‐Activated Protein Kinase (MAPK) Pathway

**Figure 3:**
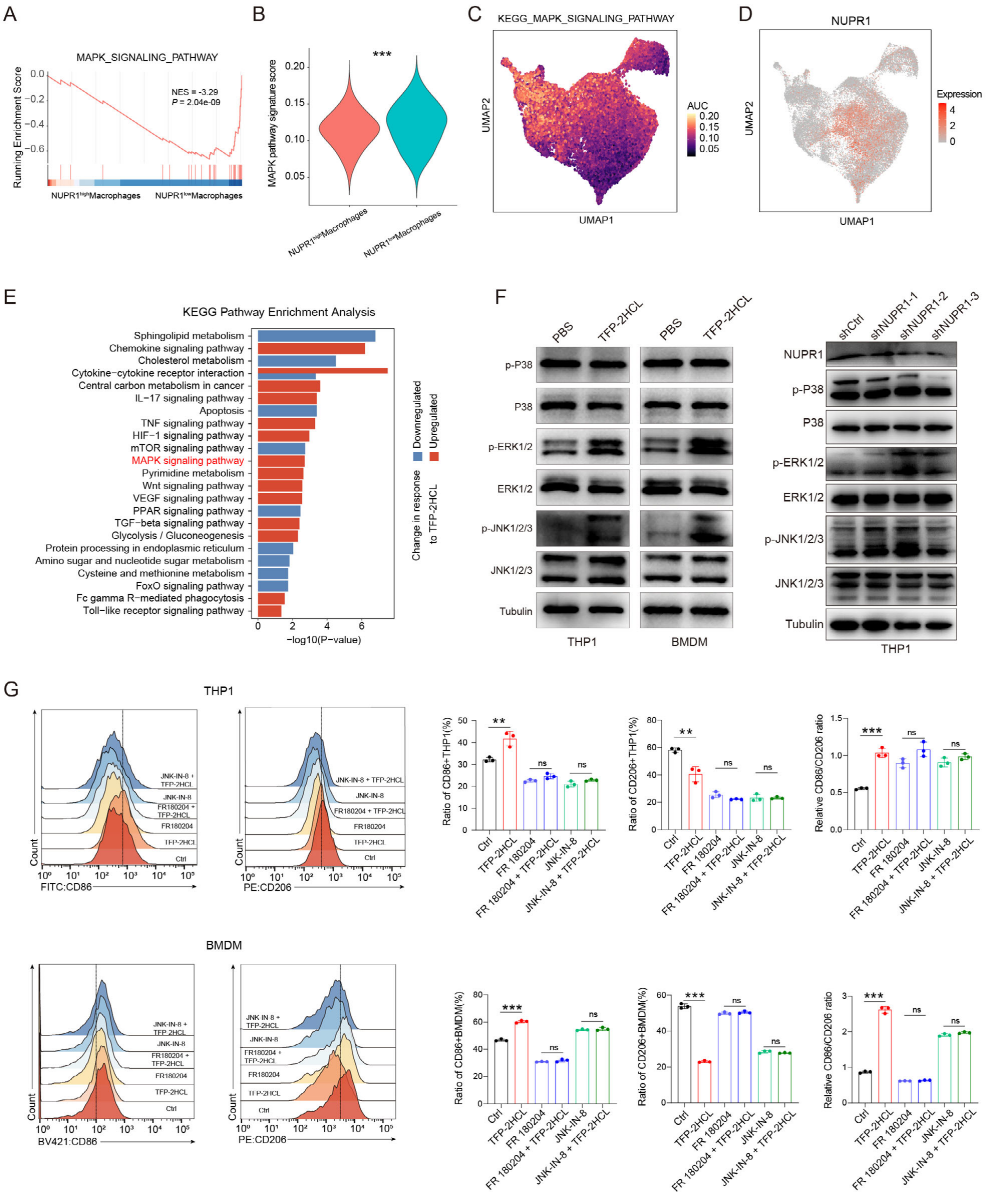
NUPR1 promotes macrophage phenotypic transition via the ERK and JNK pathway. A) GSEA analysis indicates the enrichment of the MAPK pathway in NUPR1low macrophages. B) Violin plots illustrating the MAPK pathway scores between NUPR1high and NUPR1low macrophages. C,D) UMAP plots illustrating the AUC score of the MAPK pathway and the expression of Nupr1 in integrated macrophages. E) KEGG analysis of differentially expressed genes between PBS and TFP‐2HCL treatment groups. F) Western blotting of the MAPK pathway in macrophages treated with TFP‐2HCL or transfected with shNUPR1 plasmid. G) Flow cytometric analysis of CD86 and CD206 on macrophages treated with either TFP‐2HCL (10 µm), FR180204 (50 µm), FR180204 (50 µm) + TFP‐2HCL (10 µm), JNK‐IN‐8 (20 µm), JNK‐IN‐8 (20 µm) + TFP‐2HCL (10 µm), or vehicle (*n* = 3). Results are representative of at least three independent experiments. Data are presented as mean ± SD. *n*s, not significant; * *P* < 0.05, ** *P* < 0.01, *** *P* < 0.001.

To explore the role of NUPR1 in mediating immunosuppressive macrophages polarization, we conducted GSEA analysis on integrated macrophages. Our analysis revealed a significant enrichment of the MAPK signaling pathway in NUPR1‐low macrophages (**Figure** **3**A). We then developed a MAPK pathway signature and revealed that NUPR1‐low macrophages exhibited higher MAPK pathway scores compared to NUPR1‐high macrophages (Figure 3B). UMAP visualization further demonstrated that regions with elevated NUPR1 expression corresponded to lower MAPK pathway scores (Figure 3C,D). Additionally, analysis across macrophages in the scRNA‐seq datasets demonstrated a significant negative correlation between NUPR1 expression and MAPK pathway scores (Figure S5A, Supporting Information).

Kyoto Encyclopedia of Genes and Genomes (KEGG) analysis of DEGs from RNA‐seq data of TFP‐2HCL‐treated THP1 cells revealed significant enrichment of the MAPK signaling pathway in the treated group (Figure 3E). Subsequently, ssGSEA analysis showed higher MAPK pathway signature scores in the TFP‐2HCL‐treatment group compared to the control (Figure S5B, Supporting Information). Western blotting showed increased phosphorylation levels of extracellular signal‐regulated kinase (ERK) and c‐Jun N‐terminal kinase (JNK), but not p38, in THP‐1 cells and BMDMs upon TFP‐2HCL treatment (Figure 3F). Similarly, the knockdown of NUPR1 resulted in the upregulation of phosphorylated ERK and JNK in THP1 cells (Figure 3F). Inhibition of ERK and JNK pathways with alternative inhibitors confirmed their involvement in NUPR1‐mediated immunosuppression. Flow cytometry analysis demonstrated that treatment with TFP‐2HCL induced a shift from an M2‐like to M1‐like macrophage characteristic, which was eliminated by the inhibition of ERK or JNK pathways (Figure 3G). These data suggest that NUPR1 inhibition triggers M1 macrophage polarization through the ERK and JNK signaling pathways.

### 2.4 NUPR1+ Macrophages Mediate the Exhaustion of CD8+ T Cells via ERK and JNK Pathways

**Figure 4:**
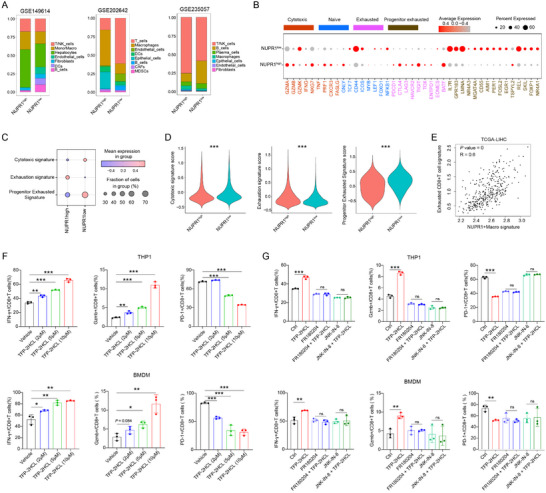
NUPR1 in macrophages promotes the exhaustion of exhausted CD8+ T cells through the ERK and JNK pathways. A) Bar chart showing the proportions of cell subpopulations in three scRNA‐seq datasets between NUPR1high and NUPR1low macrophages. B) Dot plots illustrating the expression levels of genes associated with various biological characteristics in CD8+ T cells, stratified by high and low NUPR1 expression in macrophages. C) Dot plots illustrating the scores for cytotoxic signature, exhaustion signature, and progenitor exhaustion signature in CD8+ T cells between NUPR1high and NUPR1low macrophages. D) Violin plots illustrating the score for cytotoxic signature, exhaustion signature, and progenitor exhaustion signature in CD8+ T cells between NUPR1high and NUPR1low macrophages. E) Correlation analysis showing the relationship between NUPR1+ macrophages and exhausted CD8+ T cells in the TCGA‐LIHC dataset. F) Flow cytometric analysis of IFN‐γ, Gzmb, and PD‐1 on CD8+ T cells co‐cultured with macrophages treated with TFP‐2HCL (2, 5, and 10 µm) or vehicle (*n* = 3). G) Flow cytometric analysis of IFN‐γ, Gzmb, and PD‐1 on CD8+ T cells co‐cultured with macrophages treated with either TFP‐2HCL (10 µm), FR180204 (50 µm), FR180204 (50 µm) + TFP‐2HCL (10 µm), JNK‐IN‐8 (20 µm), JNK‐IN‐8 (20 µm) + TFP‐2HCL (10 µm), or vehicle (*n* = 3). Results are representative of at least three independent experiments. Data are presented as mean ± SD. *n*s, not significant; * *P* < 0.05, ** *P* < 0.01, *** *P* < 0.001.

To elucidate the effect of NUPR1+macrophages on the TME, we re‐analyzed scRNA‐seq datasets. Samples were categorized into NUPR1‐high and NUPR1‐low groups based on the median NUPR1 expression in macrophages. Our analysis of cell proportions within the TME revealed no consistent differences in T/NK cell populations between the two groups across datasets (**Figure** **4**A). Subsequent examination of T cell‐associated gene expression revealed that genes linked to cytotoxicity and exhaustion were more highly expressed in the NUPR1‐high group, whereas genes associated with stemness and progenitor exhaustion were elevated in the NUPR1‐low group (Figure 4B; Figure S6A, Supporting Information). Constructing T cell‐related signature scores, we found that the NUPR1‐high group exhibited elevated exhaustion signature scores, while the NUPR1‐low group showed higher scores for cytotoxicity and progenitor exhaustion signature (Figure 4C,D). Previous studies demonstrated an increased proportion of exhausted CD8+ T cells leads to poor immunotherapy response,^[^ ^22^ ^]^ while a subpopulation known as progenitor‐exhausted CD8+ T cells rapidly expand and generate effector CD8+ T cells in response to immunotherapy.^[^ ^23^, ^24^ ^]^ Analysis using TCGA datasets revealed a strong positive correlation between NUPR1+ macrophages and exhausted CD8+ T cells (Figure 4E; Figure S6B, Supporting Information), supporting the role of NUPR1+ macrophages in driving CD8+ T cell exhaustion. To further investigate the effect of NUPR1+macrophages on CD8+ T cells, we established a co‐culture system involving macrophages and CD8+ T cells. Flow cytometry analysis revealed that macrophages treated with TFP‐2HCL upregulated granzyme B (Gzmb) and interferon‐gamma (IFN‐γ) expression while concurrently reducing PD‐1 expression in CD8+ T cells, suggesting that targeting NUPR1 in macrophages reversed CD8+ T cell exhaustion (Figure 4F; Figure S6C, Supporting Information). Additionally, we found that inhibition of the ERK and JNK pathways in macrophages abolished the influence of TFP‐2HCL on CD8+ T cell functionality (Figure 4G; Figure S6D, Supporting Information), underscoring the pivotal role of ERK and JNK pathways in macrophages in mediating NUPR1‐induced CD8+ T cell exhaustion.

### 2.5 Pharmacological Targeting NUPR1 Enhances the Efficacy of Anti‐PD‐1 Treatment in HCC

**Figure 5:**
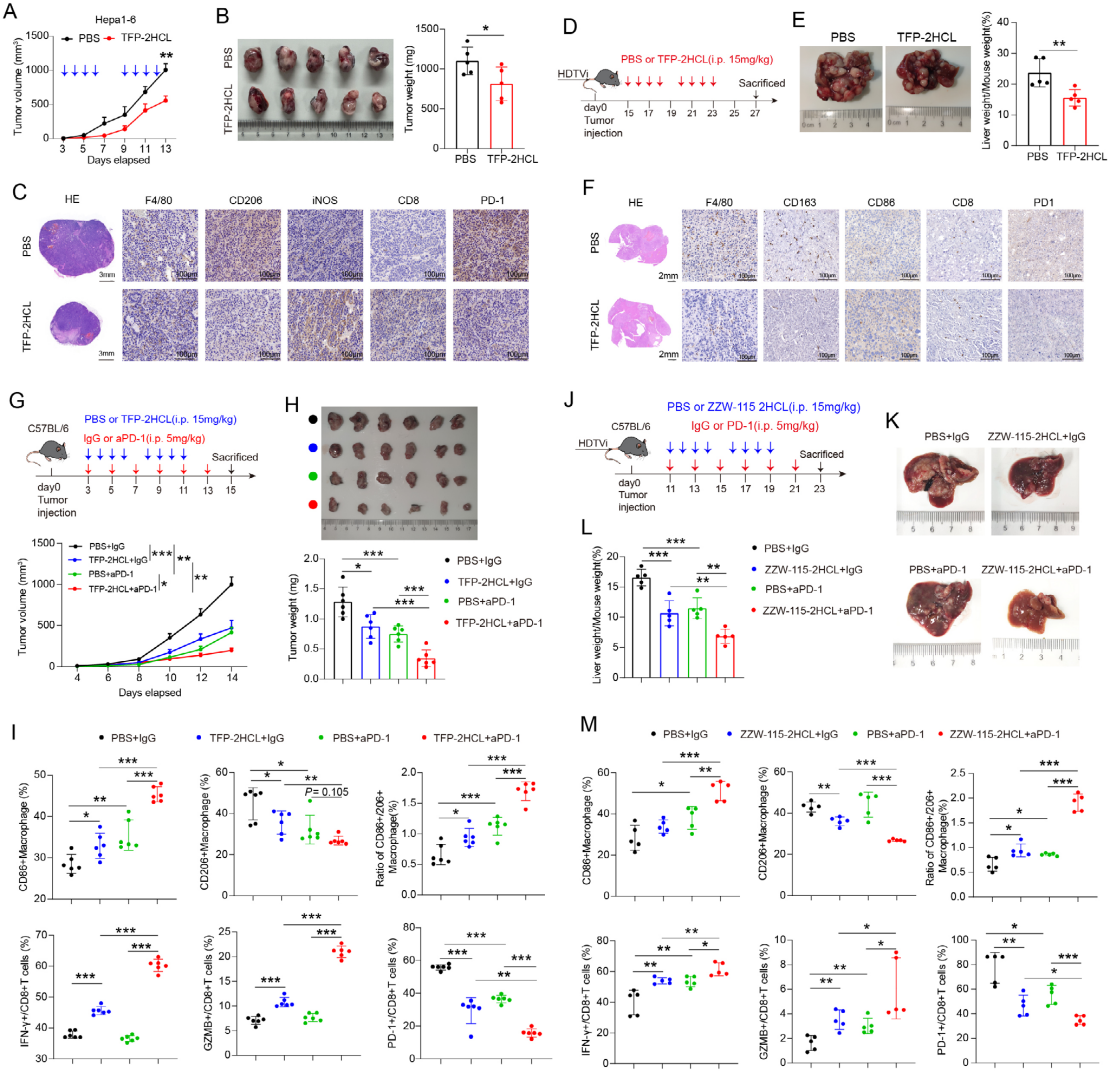
NUPR1 inhibition by TFP‐2HCL or ZZW‐115‐2HCL enhances the efficacy of anti‐PD‐1 treatment in HCC in vivo. A) Tumor growth curves of Hepa1–6 subcutaneous tumors in C57BL/6 mice treated with TFP‐2HCL or PBS (*n* = 5 each). B) Tumor weight of Hepa1–6 subcutaneous tumors in C57BL/6 mice treated with TFP‐2HCL or PBS (*n* = 5 each). C) HE and IHC were performed in TFP‐2HCL‐ or PBS‐treated subcutaneous HCC tumors. D) Workflow of the spontaneous HCC tumor treated with TFP‐2HCL or PBS. E) The left panel displays a representative image of a liver harvested from a C57BL/6 mouse with spontaneous HCC tumors on day 27. The right panel presents liver weight data from these spontaneous tumors (*n* = 5). F) HE and IHC were performed in TFP‐2HCL‐ or PBS‐treated spontaneous HCC tumors. G) Schematic representation of the treatment schedule and tumor growth curves for anti‐PD‐1 and TFP‐2HCL in C57BL/6 mice bearing HCC tumors (*n *= 5). H) Representative image of a Hepa1‐6 cell‐derived tumor and weight data harvested from C57BL/6 mice on day 15 (*n* = 6). I) Percentages of tumor‐infiltrating CD86+ macrophages, CD206+ macrophages, IFN‐γ+CD8+ T cells, GZMB+ CD8+ T cells, and PD‐1+ CD8+ T cells from subcutaneous HCC tumor (*n* = 6). J) Schematic representation of the treatment schedule for anti‐PD‐1 and ZZW‐115‐2HCL in C57BL/6 mice bearing spontaneous HCC tumors. K) Representative image of liver harvested from spontaneous tumor C57BL/6 mice on day 23 (*n* = 5). L) Bar plots show the liver weight data from these spontaneous tumors (*n* = 5). M) Percentages of tumor‐infiltrating CD86+ macrophages, CD206+ macrophages, IFNγ+CD8+ T cells, GZMB+ CD8+ T cells, and PD‐1+ CD8+ T cells from spontaneous HCC tumor (*n* = 5). Data are presented as mean ± SD. *n*s, not significant; * *P* < 0.05, ** *P* < 0.01, *** *P* < 0.001.

To evaluate the effects of NUPR1 in vivo, hepa1‐6 cells were injected subcutaneously into the axilla of mice to establish HCC tumors. Treatment with TFP‐2HCL significantly shrank tumor volume and weight (**Figure** **5**A,B). Immunohistochemistry (IHC) staining of the subcutaneous tumors revealed a decrease in CD206 expression and an increase in iNOS expression following TFP‐2HCL treatment, alongside an increase in CD8+ T cell infiltration and a reduction in PD‐1 expression (Figure 5C). To more accurately replicate the TME of HCC, we employed a hydrodynamic tail vein injection method to deliver a plasmid premixed with saline under high pressure, inducing orthotopic HCC tumors in mice (Figure 5D). Mice were euthanized at intervals starting on day 7 post‐injection to monitor the occurrence of spontaneous tumor formation in the liver. TFP‐2HCL treatment significantly diminished liver tumor burden (Figure 5E). Consistent with the subcutaneous model, IHC staining of liver tumors showed decreased CD206 expression, increased iNOS expression, elevated CD8+ T cell infiltration, and reduced PD‐1 expression (Figure 5F), suggesting that NUPR1 inhibition may restore a more immunostimulatory microenvironment conducive to tumor cell eradication.

To explore the effect of NUPR1 inhibition on PD‐1 immunotherapy, we established subcutaneous HCC tumors using Hepa1‐6 cells and initiated intraperitoneal drug administration from day 3 (Figure 5G). The results showed that both PD‐1 monoclonal antibody (mAb) and TFP‐2HCL significantly diminished tumor growth, with the combination treatment demonstrating the greatest inhibition (Figure 5G,H), with no apparent side effects (Figure S7C, Supporting Information). Subsequently, we dissociated the tumors into individual cells for flow cytometry analysis. The results demonstrated that PD‐1 mAb or TFP‐2HCL application led to a shift from M2 to M1 macrophage polarization, with the combined treatment proving most effective (Figure 5I; Figure S7A,B, Supporting Information). Additionally, TFP‐2HCL increased the proportion of IFN‐γ+CD8+ T cells and Gzmb+CD8+ T cells while decreasing the proportion of PD‐1+CD8+ T cells, thus reversing the immune microenvironment depletion. This effect was further enhanced by the combination of PD‐1 mAb and TFP‐2HCL (Figure 5I; Figure S7A,B, Supporting Information). Given the significant neuropsychiatric side effects of TFP‐2HCL, we explored the application of ZZW‐115‐2HCL, a derivative with reduced neurological effects, in a spontaneous orthotopic HCC model (Figure 5J). Treatment with ZZW‐115‐2HCL, alone or in combination with PD‐1 mAb, significantly enhanced anti‐tumor efficacy (Figure 5K,L). Flow cytometry confirmed that both PD‐1 mAb and ZZW‐115‐2HCL treatments alone reversed the immunosuppressive microenvironment, and their combination further potentiated this effect (Figure 5M; Figure S7D, Supporting Information). To further validate the functional role of NUPR1 in macrophages in an in vivo setting, we conducted an intratumoral macrophage transfer experiment. Murine BMDMs were isolated and differentiated, followed by pretreatment with either PBS or TFP‐2HCL for 48 h. The pretreated macrophages were then injected intratumorally into C57BL/6 mice bearing Hepa1–6 subcutaneous tumors (Figure S7E, Supporting Information). The results demonstrated that tumors in mice receiving TFP‐2HCL‐treated BMDMs were significantly smaller than those in the control group (Figure S7F, Supporting Information), indicating that NUPR1 inhibition in macrophages suppresses tumor growth in vivo. Subsequently, we measured the lactate content in tumor tissues and analyzed the expression levels of CD206 and NUPR1. Correlation analysis revealed a significant positive correlation between lactate levels and the expression of both CD206 and NUPR1 in the tumor tissue (Figure S7G, Supporting Information). These findings further highlight the critical role of NUPR1 in macrophage‐mediated immune suppression and tumor progression. In summary, our studies demonstrate that concurrent administration of PD‐1 mAb and pharmacological targeting of NUPR1 effectively remodel the tumor immune microenvironment, reduce macrophage‐mediated immune suppression, and promote tumor regression.

### 2.6 Tumor‐Derived Lactate Was Responsible for the Upregulation of NUPR1

**Figure 6:**
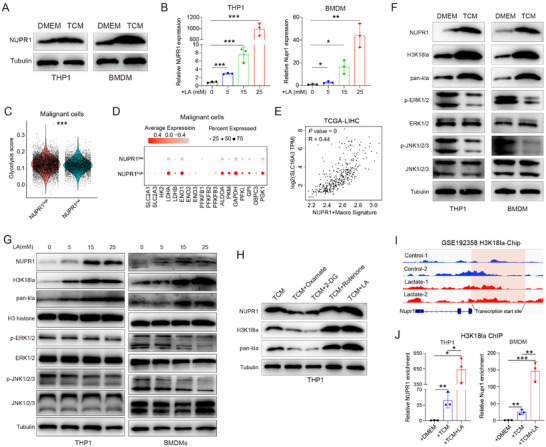
Tumor cells promote the expression of NUPR1 in macrophages via H3K18 lactylation. A) Western blotting showing the expression of NUPR1 in macrophages co‐cultured with TCM compared to controls. B) RT‐PCR analysis of NUPR1 mRNA in THP1 cells and BMDMs exposed to increasing concentrations of lactate (*n* = 3). C) Violin plot illustrating the glycolysis scores in malignant cells based on NUPR1 expression in macrophages from the GSE149614 dataset. D) Dot plot illustrating the expression of genes associated with glycolysis malignant cells based on NUPR1 expression in macrophages from the GSE149614 dataset. E) Correlation analysis between NUPR1+macrophages and SLC16A3 in the TCGA‐LIHC dataset. F) Western blotting analysis of the indicated proteins from the whole‐cell lysate of THP1 cells and BMDMs co‐cultured with TCM. G) Western blotting analysis of the indicated proteins from the whole‐cell lysate of THP1 cells and BMDMs treated with lactic acid at indicated concentrations. H) Western blot analysis of the indicated proteins in THP1 cells after co‐culture with conditioned media from tumor cells treated with metabolic inhibitors: Oxamate, 2‐DG, Rotenone, and lactic acid (LA) for 24 h. I) Genome browser tracks of ChIP‐seq signal from GSE192358 dataset at NUPR1 loci. J) H3K18la relative occupancy with NUPR1 promoter in THP1 cells and BMDMs co‐cultured with naïve, TCM, or TCM plus lactic acid for 24 h was analyzed by ChIP‐qPCR (*n* = 3). Results are representative of at least three independent experiments. Data are presented as mean ± SD. *n*s, not significant; * *P* < 0.05, ** *P* < 0.01, *** *P* < 0.001.

Next, our study investigated the underlying mechanisms driving the upregulation of NUPR1. Analysis of bulk RNA‐seq, scRNA‐seq and ST data (Figure 1) confirmed that NUPR1 was significantly higher in macrophages within the TME compared to non‐tumor tissues. This observation led us to hypothesize that tumor cells induce NUPR1 expression in macrophages. Western blotting revealed that co‐culturing macrophages with TCM upregulated NUPR1 expression in macrophages (**Figure** **6**A). Specifically, the addition of lactate significantly enhanced NUPR1 expression in macrophages (Figure 6B). Additionally, analysis of the GSE149614 dataset revealed that samples with NUPR1‐high macrophages exhibited elevated glycolysis signature scores in the epithelial cells (Figure 6C). Dot plots revealed genes associated with glycolysis were upregulated in the NUPR1‐high group (Figure 6D). Interrogation of the TCGA‐LIHC dataset identified a strong positive correlation between NUPR1+ macrophages and the lactate transporter SLC16A3 (Figure 6E). Increasing evidence indicates that lactate regulates macrophage polarization through lactylation modifications. Western blotting analysis confirmed that TCM promoted the upregulation of histone lactylation and NUPR1 expression in macrophages (Figure 6F). We have proved that NUPR1 inhibited the phosphorylation of the JNK and ERK signaling pathways. Consistent with these findings, western blotting data showed that TCM induced NUPR1 expression and inhibited the phosphorylation of the JNK and ERK pathways (Figure 6F). Additionally, the addition of lactate significantly upregulated NUPR1 expression and histone lactylation levels, while also suppressing the phosphorylation of the JNK and ERK signaling pathways (Figure 6G). Additionally, we observed that when macrophages were co‐cultured with TCM derived from tumor cells supplemented with lactate, or treated with Rotenone, which promotes glycolysis, there was a notable upregulation of NUPR1 expression and histone lactylation modifications in THP1 cells (Figure 6H). In contrast, TCM from tumor cells treated with Oxamate or 2‐Deoxy‐*D*‐glucose (2‐DG), both of which inhibit glycolysis, resulted in a suppression of NUPR1 expression and histone lactylation in THP1 cells (Figure 6H).

Li et al. conducted RNA‐seq and chromatin immunoprecipitation sequencing (ChIP‐seq) analyses on embryonic stem cells stimulated with lactate, confirming that lactate upregulated the levels of histone lactylation.^[^ ^25^ ^]^ A re‐analysis of RNA‐seq data revealed that NUPR1 expression was upregulated following lactate treatment (Figure S8, Supporting Information). Furthermore, an examination of the ChIP‐seq data revealed lactate exposure resulted in increased occupancy at the NUPR1 transcriptional start site compared to control conditions (Figure 6I). Our ChIP assays confirmed that the enhanced H3K18 lactylation induced by TCM or lactate promoted the transcriptional upregulation of NUPR1 (Figure 6J). Collectively, these findings demonstrate that tumor‐derived lactate upregulated histone lactylation levels, thereby driving the expression of NUPR1 in macrophages.

### 2.7 NUPR1+Macrophages Act as a Biomarker for Tumor Immunotherapy

**Figure 7:**
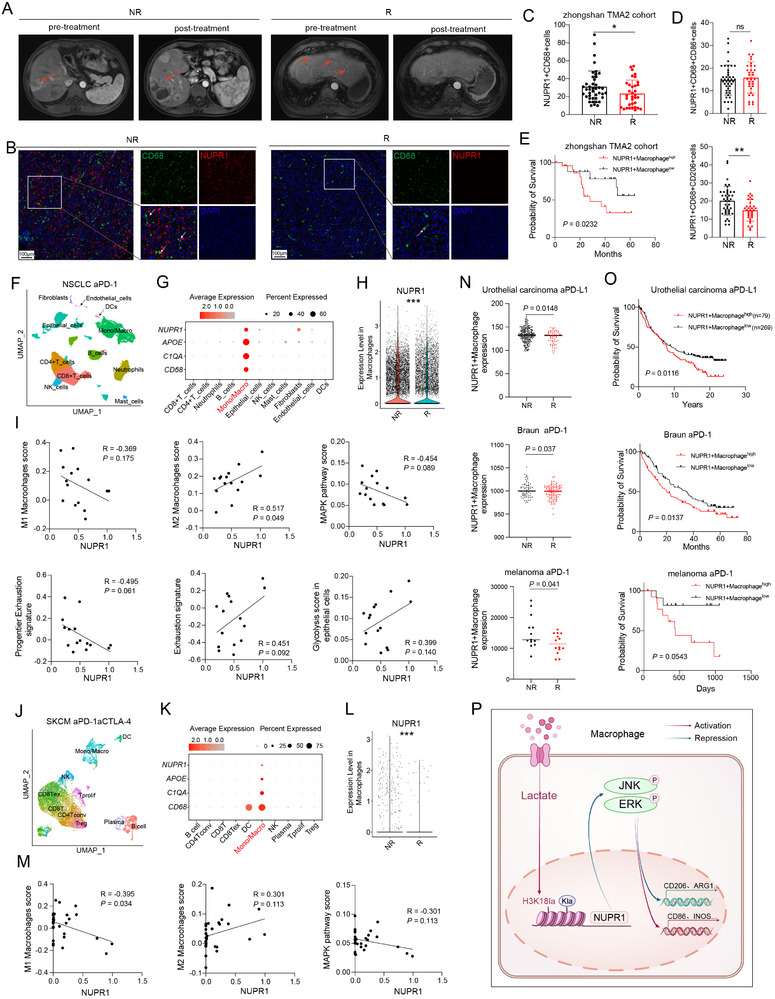
NUPR1+macrophages mediate the resistance to immunotherapy. A) Magnetic resonance imaging scans showing a responder and a non‐responder to PD‐1 mAbs before and after treatment. B) mIF images showing CD68 and NUPR1 expression in a responder and a non‐responder to PD‐1 mAb therapy. C) The number of NUPR1+CD68+cells in response patients (*n* = 33) and non‐response patients (*n* = 42) of the Zhongshan TMA2 cohort. D) The number of NUPR1+CD68+CD86+cells and NUPR1+CD68+CD206+cells in response patients and non‐response patients of the Zhongshan TMA2 cohort. E) Kaplan–Meier curves for OS of patients with HCC from the zhongshan TMA2 cohort. F) UMAP plots depicting various cell types in NSCLC tumors treated with PD‐1 mAbs from the GSE207422 dataset. G) Dot plots displaying the expression of NUPR1, C1QA, APOE, and CD68 among different cell types in the GSE207422 dataset. H) Violin plots illustrating the expression of NUPR1 in macrophages from PD‐1 mAb therapy responders and non‐responders. I) Correlation analysis of mean NUPR1 expression in macrophages with M1 macrophage score, M2 macrophage score, MAPK pathway score, progenitor exhaustion signature, exhaustion signature, and Glycolysis signature in the GSE207422 dataset. J) UMAP plots depicting various cell types in NSCLC tumors treated with PD‐1 mAbs and CTLA‐4 mAbs from the GSE120575 dataset. K) Dot plots displaying the expression of NUPR1, C1QA, APOE, and CD68 among different cell types in the GSE120575 dataset. L) Violin plots illustrating the expression of NUPR1 in macrophages from PD‐1 and CTLA‐4 mAb therapy responders and non‐responders. M) Correlation analysis of mean NUPR1 expression in macrophages with M1 macrophage score, M2 macrophage score, and MAPK pathway score in the GSE120575 dataset. N) Bar graph displaying the expression differences of NUPR1+ macrophages between responders and non‐responders in three immunotherapy datasets. O) Kaplan–Meier survival curves demonstrating that patients with HCC exhibiting high levels of NUPR1+ macrophages have poorer overall prognosis compared to those with lower expression levels in three immunotherapy datasets. P) Illustration of the proposed working model. Data are presented as mean ± SD. *n*s, not significant; * *P* < 0.05, ** *P* < 0.01, *** *P* < 0.001.

To explore the impact of NUPR1+ macrophages on immunotherapy outcomes, tumor tissues were collected from patients with HCC undergoing PD‐1 blockade at Zhongshan Hospital, affiliated to Fudan University (**Figure** **7**A). Analysis revealed a significant reduction in the presence of NUPR1+CD68+ cells in patients who responded to immunotherapy compared to those who did not (Figure 7B,C), indicating NUPR1+ macrophages contribute to resistance against HCC immunotherapy. Further subgroup analysis showed that the proportion of CD68⁺CD206⁺NUPR1⁺ cells was significantly higher in the non‐responding group compared to the responding group, whereas the proportion of CD68⁺CD86⁺NUPR1⁺ cells did not differ significantly between the two groups. This finding confirms that NUPR1 expression in M2 macrophages mediates resistance to immunotherapy (Figure 7D). Additionally, higher levels of NUPR1+CD68+ cells were associated with poorer prognosis in patients with HCC (Figure 7E).

Analysis of publicly available scRNA‐seq datasets from patients with non‐small cell lung cancer (NSCLC) treated with immunotherapy revealed that NUPR1 was predominantly expressed in macrophages, with minimal expression observed in fibroblasts (Figure 7F,G). Violin plot analysis demonstrated a significant elevation of NUPR1 expression in macrophages from non‐responsive patients, while NUPR1 expression in fibroblasts showed no discernible difference between responders and non‐responders (Figure 7H; Figure S9A, Supporting Information). NUPR1 expression in macrophages exhibited a strong positive correlation with M2 macrophages and a negative correlation with M1 macrophages and the MAPK pathway signature. It was also positively correlated with exhausted CD8+ T cells and negatively correlated with cytotoxic and progenitor exhaustion signatures. Additionally, NUPR1 expression in macrophages was positively associated with the glycolysis signature in epithelial cells (Figure 7I; Figure S9B, Supporting Information). Subsequent analysis of scRNA‐seq data from patients with skin cutaneous melanoma (SKCM) treated with a combination of PD‐1 and cytotoxic T‐lymphocyte‐associated protein 4 (CTLA‐4) mAbs revealed exclusive expression of NUPR1 in macrophages (Figure 7J,K). Similar to the NSCLC dataset, NUPR1 expression in macrophages was significantly elevated in non‐responders (Figure 7L). This cohort also exhibited NUPR1 expression in macrophages had a positive correlation with M2 macrophages and a negative correlation with M1 macrophages and the MAPK pathway signature (Figure 7M; Figure S9C, Supporting Information).

Previous research by Liu and colleagues, which integrated pan‐cancer scRNA‐seq datasets, identified apolipoprotein E (APOE)+ macrophages as playing a critical role in mediating resistance to immunotherapy across various tumor types.^[^ ^26^ ^]^ Our study revealed a strong positive correlation between NUPR1+ macrophages and APOE+ macrophages (Figure S9D, Supporting Information). Analysis of multiple bulk RNA‐seq datasets from immunotherapy‐treated patients revealed a consistent association between poor response and elevated NUPR1+ macrophage expression across several cancer cohorts, including IMvigor210,^[^ ^27^ ^]^ Braun et al.^[^ ^28^ ^]^ and Hugo et al.^[^ ^29^ ^]^ (Figure 7N). Additionally, a bulk RNA‐seq dataset from a renal cell carcinoma mouse model treated with PD‐1 therapy supported our findings, showing higher NUPR1+ macrophage expression in immunotherapy‐resistant tumors (Figure S9E, Supporting Information). Cross‐species evidence thus reinforces the role of NUPR1+ macrophages as a predictive marker for immunotherapy response. Kaplan–Meier survival analysis indicated that cancer patients with elevated NUPR1 expression in macrophages experienced significantly shorter OS (Figure 7O), underscoring the potential of NUPR1+ macrophages as a biomarker for predicting resistance to cancer immunotherapy. These data collectively confirmed that NUPR1 expressed on macrophages is associated with the immunotherapy response and patient survival in tumors treated with anti‐PD‐1. Consequently, the aforementioned findings established that NUPR1, a gene overexpressed in TAMs, is a key role in promoting M2 macrophage polarization via ERK and JNK pathways. Furthermore, this study illustrates that the lactate secreted by tumor cells induces histone modification through lactylation. This modification further enhances NUPR1 promoter activity, thus fostering an immunosuppressive TME and contributing to PD‐1 immunotherapy resistance (Figure 7P).

## 3 Discussion

Immunotherapy targeting for PD‐1 is becoming increasingly vital in the treatment of HCC. However, only a small proportion of patients benefit due to the complex regulatory mechanisms exerted by various cells within the TME. Among these cells, macrophages, which constitute a significant portion of the TME, play a crucial role in establishing an immunosuppressive environment that leads to immune evasion and resistance to immunotherapy. In this research, we uncovered a previously undescribed subpopulation of NUPR1+ macrophages through scRNA‐seq datasets. Our findings indicate that macrophages with high NUPR1 expression exhibit reduced pro‐inflammatory and T‐cell supportive capacities compared to their NUPR1 low counterparts. Furthermore, inhibition of NUPR1 effectively counteracts the immunosuppressive function of TAMs, thereby improving the response of HCC to immunotherapy.

Recent studies have demonstrated elevated expression of NUPR1 across various cancer types, correlating with poor prognosis.^[^ ^16^, ^18^, ^30^, ^31^, ^32^, ^33^ ^]^ For instance, Fan et al. revealed through in vitro and in vivo assays, as well as analysis of tumor tissues, that NUPR1 is upregulated in oral squamous cell carcinoma (OSCC) and is associated with unfavorable outcomes in patients with OSCC. NUPR1 sustains autophagic flux and lysosomal function by directly enhancing transcription factor E3 (TFE3) activity, thereby promoting both OSCC cell proliferation and metastasis.^[^ ^33^ ^]^ Similarly, Wang et al. discovered that NUPR1 mediates tamoxifen resistance in breast cancer by upregulating the gene expression related to autophagy and drug resistance.^[^ ^16^ ^]^ NUPR1 has been implicated in several biological characteristics, including ferroptosis,^[^ ^18^, ^34^ ^]^ lipogenesis,^[^ ^35^ ^]^ drug resistance,^[^ ^17^, ^36^ ^]^ and angiogenesis^[^ ^37^ ^]^ in HCC. However, these researches predominantly focused on the direct impact of NUPR1 within tumor cells, neglecting a comprehensive exploration of its roles within the TME. In our study, utilizing both human and murine scRNA‐seq datasets, we observed that NUPR1 is primarily expressed in macrophages instead of in malignant cells or other immune cells in HCC. Additionally, analysis of bulk RNA‐seq, scRNA‐seq, as well as ST data revealed that NUPR1 expression in intratumoral macrophages is significantly higher than in macrophages from adjacent tissues. Co‐culture assays with TCM demonstrated a notable upregulation of NUPR1 expression in macrophages.

In a study by Amit et al., a single‐cell RNA profiling method was utilized to monitor the temporal dynamics of circulating immune cells as they infiltrated the TME.^[^ ^20^ ^]^ The study found that within 24 h of tumor entry, macrophages transitioned into a TAM phenotype, exhibiting immunosuppressive characteristics. Reanalysis these data demonstrated that NUPR1 expression in intratumoral macrophages increased over time. This suggests a potential link between the formation of TAMs and the upregulation of NUPR1, highlighting its role in the immunosuppressive TME. Additionally, by constructing a NUPR1+ macrophage signature score, we discovered that patients with high expression of NUPR1+ macrophages experience poor prognosis. In contrast, using the median tissue expression of NUPR1 as a threshold, the prognosis did not significantly differ between patients with high and low NUPR1 expression. This highlights that NUPR1 in macrophages is a key contributor to poor prognosis in HCC. Comparable results have been reported in various other cancer types. Ren et al. demonstrated that knocking down NUPR1 in esophageal squamous cell carcinoma cells resulted in the upregulation of phosphorylated p38.^[^ ^31^ ^]^ Li et al. showed that NUPR1 knockdown in glioblastoma cells reduced the levels of phosphorylated ERK1/2 and p38 MAPK.^[^ ^38^ ^]^ In contrast, our study revealed that inhibition of NUPR1 in THP1 and BMDM cells did not significantly alter phosphorylated p38 MAPK levels; however, phosphorylated ERK and JNK levels increased significantly, underscoring the differential roles of NUPR1 across various cell types.

CD8+ T cells, also referred as cytotoxic T lymphocytes, play a crucial role in orchestrating the immune response to cancer by directly targeting and eliminating malignant cells through the secretion of cytotoxic molecules, such as perforin and granzyme. In the context of immunotherapy, particularly with immune checkpoint inhibitors like PD‐1/PD‐L1 blockades, the effective activation and expansion of CD8+ T cells are essential for achieving clinical success. However, the accumulation of exhausted CD8+ T cells within TME often correlates with resistance to immunotherapy. Our study advances this understanding by revealing that the inhibition of NUPR1 in TAMs reverses their immunosuppressive phenotype and improves their capacity to activate CD8+ T cells. This shift is evidenced by a reduction in the proportion of exhausted PD‐1+ CD8+ T cells and an increase in the population of cytotoxic CD8+ T cells within the TME. Furthermore, in vivo assays utilizing two murine HCC models demonstrated that pharmacological inhibition of NUPR1 significantly suppressed tumor growth, accompanied by increased M1 macrophage polarization and enhanced CD8+ T cell activity. Notably, the combination of NUPR1 inhibition with anti‐PD‐1 therapy resulted in a synergistic enhancement of therapeutic efficacy. Moreover, previous research has underscored the significance of a subset of exhausted CD8+ T cells, termed progenitor exhausted CD8+ T cells, which are closely associated with favorable responses to immunotherapy. Our scRNA‐seq data revealed that patients exhibiting high NUPR1 expression in macrophages displayed significantly lower progenitor‐exhausted CD8+ T cell signature scores compared to those with low NUPR1 expression, suggesting a potential mechanism through which NUPR1 targeting augments anti‐PD‐1 therapy. These results suggest that targeting NUPR1 in macrophages could enhance the efficacy of ICB therapy in HCC. By modulating the immunosuppressive phenotype of macrophages, we can potentially overcome resistance to immunotherapy and improve patient outcomes. Additionally, our study identifies a strong correlation between macrophage‐specific NUPR1 expression and poor therapeutic outcomes in patients with HCC undergoing PD‐1 blockade. mIF staining of tissue samples from patients with HCC at Zhongshan Hospital revealed a markedly higher prevalence of NUPR1+CD68+ macrophages in the non‐responsive group compared to the responsive group. Liu et al. conducted an integrative analysis of macrophages across multiple tumor types using single‐cell RNA sequencing data and demonstrated that APOE+ macrophages are closely associated with resistance to immunotherapy.^[^ ^26^ ^]^ Our research reveals a strong correlation between NUPR1+ macrophages and APOE+ macrophages, underscoring the potential involvement of NUPR1 in shaping the immunosuppressive phenotype that underlies poor therapeutic response.

TFP‐2HCL, originally developed as an antipsychotic drug, has recently shown promise as a therapeutic agent in cancer treatment due to its potential to inhibit NUPR1, a protein implicated in tumor progression.^[^ ^39^, ^40^, ^41^ ^]^ Studies have highlighted trifluoperazine as a promising candidate for cancer therapy, demonstrating its multifaceted anticancer properties. Our research indicates that targeting NUPR1 in macrophages with trifluoperazine reverses the immunosuppressive characteristics of TAMs. Additionally, combining trifluoperazine with anti‐PD‐1 therapy significantly improves the immune‐activating TME in HCC, enhancing the efficacy of the treatment. However, trifluoperazine has been reported to exhibit strong neuropsychiatric side effects in vivo, which limits its clinical application. To address this issue, researchers have engineered ZZW‐115, a derivative compound that exhibits minimal neuropsychiatric effects. Zhang et al. demonstrated the inhibitory effects of ZZW‐115 on HCC.^[^ ^18^ ^]^ Our study extends these findings by confirming that ZZW‐115 can improve the TME by inhibiting NUPR1 in macrophages and enhancing the efficacy of anti‐PD‐1 therapy, thereby advancing the understanding of ZZW‐115′s role in HCC treatment.

Since its discovery by Yingming Zhao in 2019,^[^ ^42^ ^]^ lactylation has garnered significant attention for its detrimental roles in tumor biology. Current research indicates that lactylation primarily exerts its effects through two mechanisms: directly modifying proteins to regulate their function^[^ ^43^ ^]^ and activating transcription via histone lactylation.^[^ ^44^ ^]^ In the context of macrophages, studies have shown that lactylation induces M2 macrophage markers through histone modifications and promotes the formation of immunosuppressive macrophage subsets by upregulating immunosuppressive genes.^[^ ^45^ ^]^ Besides, Studies have shown that methylation and acetylation modifications are more prevalent in M1 macrophages, while lactylation modifications are increased in M2 macrophages.^[^ ^45^ ^]^ In this study, we identified that lactate increases NUPR1 expression in macrophages while concurrently reducing the levels of phosphorylated ERK and JNK, indicating that the lactate‐NUPR1‐ERK/JNK axis plays a pivotal role in modulating macrophage‐mediated immunosuppression. Furthermore, ChIP assays confirmed that histone lactylation induced by lactate activates NUPR1 transcription in macrophages. Multi‐omics analyses revealed that NUPR1 expression is significantly higher in TAMs compared to macrophages from normal tissues. Patients with elevated NUPR1 expression in macrophages also exhibited higher glycolysis scores in tumor cells than those with lower NUPR1 expression. Subsequent experiments demonstrated that tumor‐derived supernatants upregulate NUPR1 expression in macrophages while inhibiting tumor cell glycolysis markedly reduced the supernatant's ability to induce NUPR1 expression in macrophages. These findings underscore the metabolic interplay between tumor cells and macrophages in fostering an immunosuppressive microenvironment.

In summary, our findings demonstrate that high NUPR1 expression within the HCC TME promotes the development of a highly immunosuppressive subset of macrophages. This subset is characterized by enhanced angiogenesis, T‐cell exhaustion, and diminished inflammatory responses. Our analysis has identified a previously unrecognized population of macrophages marked by elevated NUPR1 expression, establishing its clinical relevance in predicting the prognosis of patients with HCC. Furthermore, our findings position NUPR1 as a promising biomarker for predicting patient responses to ICB therapy. Targeting NUPR1 may disrupt the immunosuppressive TME orchestrated by intratumoral macrophages, thereby enhancing CD8+ T cell‐mediated immune response.

In conclusion, our study underscores the critical role of NUPR1 in establishing the immunosuppressive microenvironment of HCC for the first time. Targeting NUPR1 may provide a novel strategy to enhance the efficacy of immunotherapy and improve outcomes for patients with HCC.

### 3.1 Limitations of the Study

This study has several limitations that merit consideration. First, while we employed a selective inhibitor of NUPR1 for in vivo experiments, NUPR1 is also expressed at varying levels in other immune cells and tumor cells. Consequently, the effects of NUPR1 within macrophages on the TME necessitate more precise experimental approaches to isolate its specific role. Additionally, beyond tumor cells, stromal cells such as fibroblasts also contribute to the acidic microenvironment, potentially influencing NUPR1 regulation in macrophages. The specific regulatory effects of stromal cells on NUPR1 within macrophages require further investigation. Besides, while lactate can directly enhance the expression of genes like ARG1 through histone lactylation, the extent to which lactate influences macrophage immunosuppressive functions via NUPR1 remains to be fully elucidated.

## 4 Experimental Section

### Publicly Available Data Collection and Processing

Publicly available transcriptome data were systematically collected from cancer patients to study tumor response to various therapies. Bulk RNA‐seq data, along with clinical information, were acquired from several cohorts including GSE54236, GSE191252, GSE14520 on the gene expression omnibus (GEO), and LIHC cohorts from The Cancer Genome Atlas (TCGA) and the international cancer genome consortium (ICGC). Additionally, datasets involving patients who received immunotherapy, such as those from the IMvigor210, Braun et al., and Hugo et al., were obtained from the tumor immune dysfunction and exclusion database (TIDE; http://tide.dfci.harvard.edu/). Patients demonstrating partial response (PR) or complete response (CR) were categorized as responders, while those with progressive disease (PD) or stable disease (SD) were considered non‐responders. Prior to analysis, all transcriptome data were normalized using the “limma” package in R. The “clusterProfiler” R package was utilized to convert Ensemble IDs to gene symbols, ensuring accurate gene identification across different datasets. For single‐cell RNA‐seq (scRNA‐seq) analysis, data from GSE149614, GSE202642, GSE235057, GSE232182, GSE216805, and GSE232040 were downloaded from the GEO database. Datasets pertaining to NSCLC with accession number GSE207422 and SKCM with accession number GSE120575 that underwent immunotherapy were sourced from the TISCH2 database (http://tisch.comp‐genomics.org/). Spatial transcriptomics (ST) data were sourced from GEO under the accession number GSE238264, providing high‐resolution spatial context to the transcriptional activity within tumor tissues. ChIP‐Seq data for further epigenomic analysis were sourced from GEO under the accession number GSE192358.

### Clinical Tissue Samples

Two tissue microarrays (TMAs) comprising hepatocellular carcinoma (HCC) specimens were used for analysis. TMA1 included a total of 464 samples, consisting of 239 tumor tissues and partially paired adjacent normal tissues, collected from patients who underwent surgical resection at Zhongshan Hospital, Fudan University, between March 2010 and December 2010. None of the patients had received prior anticancer therapy. Clinical follow‐up was conducted until December 2015. TMA2 consisted of 75 pre‐treatment biopsy specimens from patients with HCC scheduled to receive anti‐PD‐1 immunotherapy (33 responders and 42 non‐responders) at Zhongshan Hospital, Fudan University, between September 2017 and December 2018. Biopsies were performed under computed tomography (CT) guidance prior to the initiation of anti‐PD‐1 treatment. Therapeutic response was monitored bi‐monthly using magnetic resonance imaging based on the iRECIST criteria.^[^ ^46^ ^]^ Treatment responses were categorized as follows: patients achieving immune complete response or immune partial response were considered responders; those with immune stable disease, immune unconfirm progressive disease, or immune confirm progressive disease were categorized as non‐responders. Ethical approval for this study was obtained from the Research Ethics Committee of Zhongshan Hospital (Approval No. B2021‐248), and written informed consent was obtained from all participants.

### Cell Lines

The human HCC Huh7 cells and murine HCC Hepa1‐6 cells were obtained from the Liver Cancer Institute of Fudan University‐affiliated Zhongshan Hospital. THP1 cells were acquired from the Chinese Academy of Sciences. Huh7 and Hepa1‐6 cells were cultured in Dulbecco's modified Eagle's medium (DMEM, KFMI12800N, KeyGEN BioTECH, Jiangsu, China) medium supplemented with 10% fetal bovine serum (FBS, F8318, Sigma, MO, USA), 1% penicillin and streptomycin (C0222, Beyotime, Shanghai, China), while THP1 cells were cultured in RPMI‐1640 (C11875500CP, Gibco, FL, USA) medium supplemented with 10% FBS, 1% penicillin and streptomycin.

### Single‐Cell RNA Sequencing (scRNA‐Seq) Analyses

For scRNA‐seq data analysis, raw count matrices were first processed using the Seurat R package (version 4.0.1). Stringent quality control measures were implemented, including the removal of doublets, employing DoubletFinder (version 2.0.4). Following quality control, data normalization was conducted, and batch effects were addressed across samples using the Harmony algorithm (version 1.0). To address potential cell cycle effects, scores for the S and G2/M phases were regressed out, which were calculated using the CellCycleScoring function of Seurat. For visualizing cell clusters and subpopulations, uniform manifold approximation and projection (UMAP) was utilized, leveraging the top principal components identified during the analysis. Cell subgroups were annotated through both automatic and manual methods to enhance accuracy. The SingleR package was utilized for initial annotations, which were further refined manually using established literature and database‐derived cell markers. All statistical analyses and clustering operations were conducted using R software (version 4.1.3), ensuring rigorous data processing and integrity of the analyses. Signature scoring was conducted with the AddModuleScore function in Seurat. To define the NUPR1⁺ macrophage signature, macrophages were first extracted from the three scRNA‐seq datasets of patients with HCC. Cells were then stratified into NUPR1‐high and NUPR1‐low macrophages based on the median NUPR1 expression. DEGs between these two groups were identified, followed by filtering out genes that were not specifically enriched in monocytes and macrophages. Only genes with preferential expression in monocytes/macrophages compared to other cell types were retained, resulting in a final set of 30 NUPR1⁺ macrophage signature genes. To estimate the presence of NUPR1+macrophages in bulk RNA‐seq data, the average expression of these 30 signature genes was calculated, which was used as the NUPR1⁺ macrophage signature score. The list of genes for the corresponding signature is detailed in Supporting Information.

### Spatial Transcriptome (ST) Dataset Analysis

For ST data, raw count matrices were processed. Quality control and alignment of the data were performed with Space Ranger software (version 1.2.0). Normalization was conducted with the SCTransform function within Seurat to correct for technical variations and cell‐specific biases. This normalization specifically targeted cells displaying more than 300 spatial features and mitochondrial gene content less than 30%, ensuring only high‐quality transcriptomic profiles were retained for analysis. SpatialFeaturePlot from the Seurat was employed to visualize the expression patterns of indicated markers.

### Analysis of Transcriptomic Data Using GEPIA2

Differential expression and prognostic analyses of NUPR1 in adjacent and normal tissues across LAML, LGG, TGCT, and THYM datasets were performed using the GEPIA2 database. Correlation analyses involving NUPR1+ macrophages and immunological markers such as LGALS9, SIRPA, CD274, and SLC16A3 were conducted for the LIHC dataset. Additionally, relationships between NUPR1+ macrophages and exhausted CD8+ T cells were evaluated across LIHC, BLCA, COAD, and PRAD datasets. The associations between NUPR1+ and APOE+ macrophages were analyzed in BRCA, COAD, LIHC, LUSC, and PAAD datasets.

### Isolation of BMDM

Male C57BL/6 mice aged 5–6 weeks were euthanized and their tibiae and fibulae were harvested. The bone marrow was flushed out and the resulting cell suspension was filtered through a 70 µm cell strainer to remove debris. After lysing red blood cells, the remaining cells were resuspended in complete DMEM and cultured with 40 ng mL^−1^ macrophage colony‐stimulating factor (m‐CSF, abs04383, Absin, Shanghai, China) for 7 days to differentiate into bone marrow‐derived macrophages (BMDMs).

### Tumor‐Associated Macrophages (TAMs)

THP1 cells were adjusted to a concentration of 5 × 10^−5^ cells mL^−1^ and cultured with 320 nm phorbol 12‐myristate 13‐acetate (PMA, S1819, Beyotime) to polarize into M0 macrophages after 24 h. The supernatant from Huh7 cells was harvested and clarified by filtration through a 0.22 µm membrane to remove cell debris and then mixed with RPMI 1640 medium at a 1:1 ratio. This mixture was added to M0 macrophages for 24 h to induce TAMs. For BMDM, the filtered supernatant of Hepa1‐6 cells was mixed with DMEM at a 1:1 ratio and added to BMDM for 24 h to polarize the TAMs.

### RNA Extraction and Quantitative Real‐Time Polymerase Chain Reaction (qRT‐PCR)

Total RNA was isolated using an RNA extraction kit (R0027, Beyotime) and subsequently converted to cDNA with a cDNA synthesis kit (11120ES60, Yeasen, Shanghai, China). qRT‐PCR analyses were performed using an ABI 7900HT Real‐Time PCR system (Life Technologies, Waltham, MA, USA) with SYBR Green Master Mix (11202ES08, Yeasen). Each 20 µL reaction contained 1 µL of cDNA, 1 µL of both forward and reverse gene‐specific primers, 10 µL of SYBR Green Master Mix, and 8 µL of nuclease‐free water. β‐actin was used as the internal control, and data were analyzed using the 2^−ΔΔCT^ method. The specific primers employed are detailed in Table S2 (Supporting Information).

### Protein Extraction and Western Blotting

For protein extraction, cells were lysed using cell lysis buffer (P0013B, Beyotime) containing phenylmethylsulfonyl fluoride (PMSF, ST506, Beyotime) and a cocktail of protease and phosphatase inhibitors (P1048, Beyotime). Protein samples(10 µL per well) were separated by SDS‐PAGE and transferred onto PVDF membranes (IPVH00010, Millipore, MA, USA). Membranes were blocked using a quick blocking solution (P0252, Beyotime) and incubated overnight at 4 °C with primary antibodies detailed in Table S3 (Supporting Information). After washing, they were treated with HRP‐conjugated secondary antibodies (RS0001 or RS0002, ImmunoWay, CA, USA) for 1 h at room temperature (≈25 °C.). Protein detection was performed using a chemiluminescent HRP substrate (P10100, NCM Biotech, Suzhou, China) and captured with an ECL imaging system (Tanon, Shanghai, China). The specific antibodies employed are listed in Table S3 (Supporting Information).

### ChIP

ChIP was conducted using the SimpleChIP Plus Enzymatic Chromatin IP Kit (9005, Cell Signaling Technology, MA, USA) according to the manufacturer's protocol. After formaldehyde cross‐linking, cells were lysed and chromatin was fragmented using a combination of enzymatic digestion and sonication to achieve DNA fragments ranging from 200 to 1000 base pairs. The fragmented chromatin was then incubated with specific antibodies at 4 °C with gentle agitation. Antibody‐bound complexes were captured with magnetic beads. Following several wash steps, the cross‐links were reversed to release the bound DNA, which was subsequently purified. The purified DNA was subjected to qPCR to assess the presence of specific genomic regions. The specific primers employed are detailed in Table S2 (Supporting Information).

### Flow Cytometry

Tumor tissues were initially dissected into 1 mm^3^ pieces and then placed in 8 ml of DMEM culture medium with 0.1% type 4 Collagenase (C5138, Sigma, MO, USA), 0.05% Hyaluronidase (H3506, Sigma), and 0.01% Deoxyribonuclease (DN25, Sigma). The solution was shaken at 37 °C for 30 min. After centrifugation and removal of the supernatant, the cells were resuspended in DMEM to achieve a final concentration of 1 × 10^−7^ cells mL^−1^. Cells were incubated with fixable viability dye and anti‐CD16/CD32 mAbs before surface labeling with antibodies. Subsequent steps included incubation with pre‐conjugated surface antibodies, permeabilization, and intracellular antibody incubation. Flow cytometric analysis was then carried out using a FACSAria III flow cytometer (BD Biosciences, CA, USA) after two washes and filtration through a 70 µm cell strainer. Data were collected and subsequent analysis was conducted using FlowJo software (Tree Star, OR, USA). The antibodies performed in this research are detailed in Table S3 (Supporting Information).

### Animals and In Vivo Assays

6‐week‐old C57BL/6 male mice were utilized in the study. For the subcutaneous tumor model, 5 × 10^−5^ Hepa1‐6 cells were mixed in 150 µL of PBS and injected into the right armpit of the mice. Starting from the third day, the condition of the mice was monitored, and the tumor size was documented using the formula: tumor size = length × width squared × 1/2. In the case of the spontaneous tumor model, the mouse's tail was immobilized, and then 2 ml of physiological saline containing 10 µg c‐Myc plasmid, 10 µg Ctnnb‐N90 plasmid, and 10 µg SB13 transfection plasmid was swiftly injected into the mouse's tail vein under high pressure within 5 to 7 s. The mice were euthanized between days 7 to 10, and their livers were extracted to check for the presence of spontaneous tumors. Upon reaching the desired experimental outcomes or when the tumor weight reached 10% of the mouse's body weight, the mice were humanely euthanized by rapid cervical dislocation under anesthesia, and the subcutaneous tumors or livers were excised for photography, weighing, and documentation. For the macrophage transfer model, subcutaneous HCC tumors were first established as described above. On days 5, 8, and 11 after tumor inoculation, mice received intratumoral injections (i.t.) of BMDMs pretreated with PBS or TFP‐2HCl for 48 h. The number of injected BMDM cells was normalized to 1 × 10^−4^ cells mm^−3^ of tumor volume. Tumors were collected on day 15 for downstream analyses.

Drug treatment commenced either when the subcutaneous tumor attained a volume of 5 mm^3^ or when spontaneous tumors became apparent during the examination. Each mouse received 200 µg of PD‐1 monoclonal antibody (BE0146, Bio X cell, CA, USA) or isotype control IgG antibody (BE0089, Bio X cell) via intraperitoneal injection every 3 days for a total of four injections. TFP‐2HCL (HY‐B0532A, MCE, NJ, USA) or ZZW‐115‐2HCL (HY‐111838A, MCE) was delivered at a concentration of 15 mg kg^−1 ^ by intraperitoneal injection, administered four times a week for two consecutive weeks. All animal procedures were carried out in compliance with the guidelines set forth by the Shanghai Medical Laboratory Animal Protection Committee and approved by the Animal Experiment Ethics Committee of Zhongshan Hospital, Fudan University (Approval No. 2020–133).

### Multiplex Immunofluorescence (mIF) Analysis

Slides were first deparaffinized in xylene and then rehydrated through a series of ethanol washes. Antigen retrieval was carried out with citrate buffer, and subsequent blocking was performed using 5% bovine serum albumin (BSA, ST025, Beyotime). The slides were treated with a mixture of primary antibodies at 37 °C for 1 h in a controlled humidity environment. Corresponding secondary antibodies conjugated with Alexa Fluor dyes were applied, and unbound antibodies were washed off using citrate buffer. For nuclear staining, DAPI (D1306, Thermo Fisher Scientific) was used and incubated for 10 min at 37 °C in a dark environment. Following staining, slides were mounted and imaged using a fluorescence microscope (Olympus, Tokyo, Japan). Fluorescence intensity was quantified with ImageJ software, and further analysis was carried out using CaseViewer software (Biossci, Hubei, China). Statistical analyses were performed with GraphPad Prism.

### Immunohistochemistry (IHC) Analysis

Paraffin‐embedded tissue sections were initially deparaffinized with xylene and then rehydrated through a series of ethanol washes. Antigen retrieval was performed by heating the slides in citrate buffer at a sub‐boiling temperature for 15 min. To minimize non‐specific binding, the slides were blocked with 5% BSA for 60 min. Primary antibody incubation was carried out overnight at 4 °C. Following this, slides were treated with HRP‐conjugated secondary antibodies for 1 h at 37 °C. Color development was achieved using a DAB kit (Gene Tech, Shanghai, China), and nuclear staining was performed with hematoxylin. The stained slides were examined and imaged with a microscope (Olympus) or analyzed using CaseViewer software (3DHISTECH, Budapest, Hungary).

### Short Hairpin RNA (shRNA) Transfection

THP1 cells stimulated with PMA were seeded to reach ≈70% confluence. Transfection was performed by adding a pre‐mixed solution of shRNA plasmids and Lipofectamine 8000 (C0533, Beyotime) directly to the culture medium, ensuring thorough mixing to facilitate efficient gene delivery. Following a 24‐hour incubation period, the culture medium was exchanged to eliminate residual transfection reagent and cellular debris. To confirm the efficacy of RNA interference, the expression of genes was measured one day after transfection using qPCR, while protein expression was analyzed two days later. Details of the shRNA sequences used are provided in Table S4 (Supporting Information).

### Isolation of CD8+ T Cells and Co‐Culture Assay

CD8+ T cells were isolated from the peripheral blood of healthy human donors using the EasySep Direct Human T Cell Isolation Kit (19 661, STEMCELL Technologies, WA, USA) according to the manufacturer's instructions. The isolated human CD8+ T cells were then cultured in CTS AIM V SFM medium (A3021002, Gibco) supplemented with ImmunoCult Human CD3/CD28 T cell activator (10 971, STEMCELL Technologies) and recombinant human IL‐2 (78 220, STEMCELL Technologies) for 48 h. Mouse CD8+ T cells were isolated from the spleens of wild‐type C57BL/6 mice using the CD8 T Cell Isolation Kit (480 035, BioLegend, CA, USA) in accordance with the manufacturer's protocols. These cells were preactivated by seeding onto culture plates precoated with 2 µg mL^−1^ anti‐CD3 (100 340, BioLegend) and 2 µg mL^−1^ anti‐CD28 antibodies (102 116, BioLegend). The mouse CD8+ T cells were then cultured in RPMI 1640 medium supplemented with 10% FBS, 100 U mL^−1^ IL‐2 (212‐12, Peprotech, NJ, USA), 2 mm *L*‐glutamine (0 7100, STEMCELL Technologies), 50 µm β‐mercaptoethanol (GNM21985‐1, GENOM Biotech, Jiangsu, China), 1 mm sodium pyruvate (C0331, Beyotime), 100 µm MEM non‐essential amino acids (0 7600, STEMCELL Technologies), and 10 mm HEPES (0 7200, STEMCELL Technologies) for 48 h. Macrophages were pretreated with TFP‐2HCl for 48 h, followed by medium replacement to remove residual drug before introducing CD8+ T cells. Following pre‐activation, both human and mouse CD8+ T cells were co‐cultured with macrophages at a 1:1 ratio for an additional 48 h. After co‐culture, CD8+ T cells were analyzed for the expression of granzyme B (GZMB), interferon‐γ (IFN‐γ), and PD‐1 to assess their activation and exhaustion status.

### Enzyme‐Linked Immunosorbent Assay (ELISA) Analysis

ELISA assays were conducted following the manufacturer's guidelines. Murine blood samples were allowed to clot at ambient temperature (≈25 °C), then centrifuged at 4 °C and 10 000 × *g* for 10 min to obtain the serum. The levels of alanine aminotransferase (ALT) and aspartate aminotransferase (AST) in the serum were quantified using specific assay kits for mouse ALT (R01502, Rayto, Shenzhen, China) and AST (R01702, Rayto), respectively. All measurements were conducted using the Chemray 240 automatic biochemical analyzer (Rayto), ensuring precise data acquisition.

### Hematoxylin and Eosin (HE) Staining

Tumor tissue was fixed, embedded in paraffin, and sectioned. The sections were placed in a 60 °C oven for 4–6 h to remove any residual paraffin. After deparaffinization, the sections underwent a series of xylene immersions to clear the tissue, followed by rehydration through a graded series of alcohol solutions. Staining was carried out sequentially using hematoxylin to visualize cell nuclei, followed by eosin to highlight the cytoplasm and connective tissue, thereby enhancing the contrast. After staining, sections were dehydrated through an ascending alcohol series and cleared in xylene. The prepared slides were examined under a light microscope for histopathological evaluation.

### GSEA and KEGG Analysis

Gene sets were sourced from the Molecular Signatures Database (MSigDB) (https://www.gsea‐msigdb.org/gsea/msigdb/). GSEA was performed using the GSEA software (Broad Institute, https://www.broadinstitute.org/gsea/). For KEGG pathway analysis, the “clusterProfiler” R package was employed to annotate the genes. A threshold for statistical significance was established at *P* < 0.05.

### RNA‐Sequencing (RNA‐Seq)

Total RNA was isolated from THP1 cells using TRIzol reagent (15 596 026, Invitrogen, Carlsbad, CA, USA), followed by quality assessment and quantification. mRNA was enriched using oligo(dT) magnetic beads, and libraries were prepared following the protocol of the NEBNext Ultra RNA Library Prep Kit for Illumina (New England Biolabs, USA). Sequencing was carried out with the Illumina NovaSeq platform. The quality of sequence data was assessed with FastQC, and alignment to the human reference genome GRCh38 was performed using HISAT2 (https://ccb.jhu.edu/software/hisat2/index.shtml). Gene expression was quantified using the Fragments Per Kilobase of exon per Million mapped reads (FPKM) method, and differential expression analysis was conducted using the DESeq2 R package, defining significance as a *P*‐value < 0.05 and log2 (|fold change|) > 1.

### Lactate Production

Tumor tissues of equal mass (≈0.1 g) were collected for analysis. Lactate levels were quantified using a Lactic Acid Content Assay Kit (D799099, Sangon Biotech, Shanghai, China) following the manufacturer's instructions. After sample processing, the precipitate was dissolved in 200 µL of absolute ethanol, and absorbance was measured at 570 nm using a microplate reader. Lactate concentrations were calculated based on a standard curve and normalized to total protein content to control for sample variability.

### Statistical Analysis

The numbers of individual animals or samples used per group or independent experiments are described in each individual Figure panel. Each dot corresponds to one mouse or a sample unless otherwise noted. Statistical analyses were conducted using R software (V.4.1.3) or GraphPad Prism (version 8, San Diego, CA, USA). Group differences were assessed using either the student's *t*‐test or the Mann–Whitney *U*‐test, as appropriate based on data distribution. Differences in categorical variables were assessed with the chi‐square test or Fisher's exact test, depending on the sample size and expected frequencies. Survival curves were generated with the Kaplan–Meier method and compared with the log‐rank test. Independent prognostic factors were identified through Cox proportional hazards regression analysis. Data were presented as mean ± standard deviation (SD), and statistical significance was denoted as follows: *n*s, not significant; *P < 0.05, **P < 0.01, ***P < 0.001. In all cases, significance was defined as *P* < 0.05.

## Conflict of Interest

The authors declare no conflict of interest.

## Author Contributions

J.C., P.Z., and Y.C. contributed equally to this work. J.C., P.Z., Y.C., and Z.D. designed the study. J.C., P.Z., and Y.C. conducted the majority of the experiments. G.Z. and S.C. assisted in molecular biology and function analysis performed in vivo. L.S. analyzed the bioinformatic data. J.D. and B.W. collected tissue samples and data. Y.C. and Z.D. did the statistical analyses. J.C., P.Z., G.Z., S.C., and W.D. prepared Figures, reviewed the results, interpreted data, and wrote the manuscript. Z.D., Y.Y., J.F., and J.Z. contributed to study supervision. Z.D. and Y.Y. provided financial support. All authors have made an intellectual contribution to the manuscript and approved the submission.

## Supporting information



Supporting Information

Supplemental Table 1

## Data Availability

The data that support the findings of this study are available from the corresponding author upon reasonable request.
