# Supplementary material for: Lactylation‐Driven NUPR1 Promotes Immunosuppression of Tumor‐Infiltrating Macrophages in Hepatocellular Carcinoma
Source: Adv Sci (Weinh). 2025 Apr 30;12(20):2413095. doi: 10.1002/advs.202413095 (PMC12120759; doi:10.1002/advs.202413095)
Supplement: Supplementary file 1 — Supporting Information [file ADVS-12-2413095-s001.docx]

**Supplementary Files**

**Supplementary Figures**


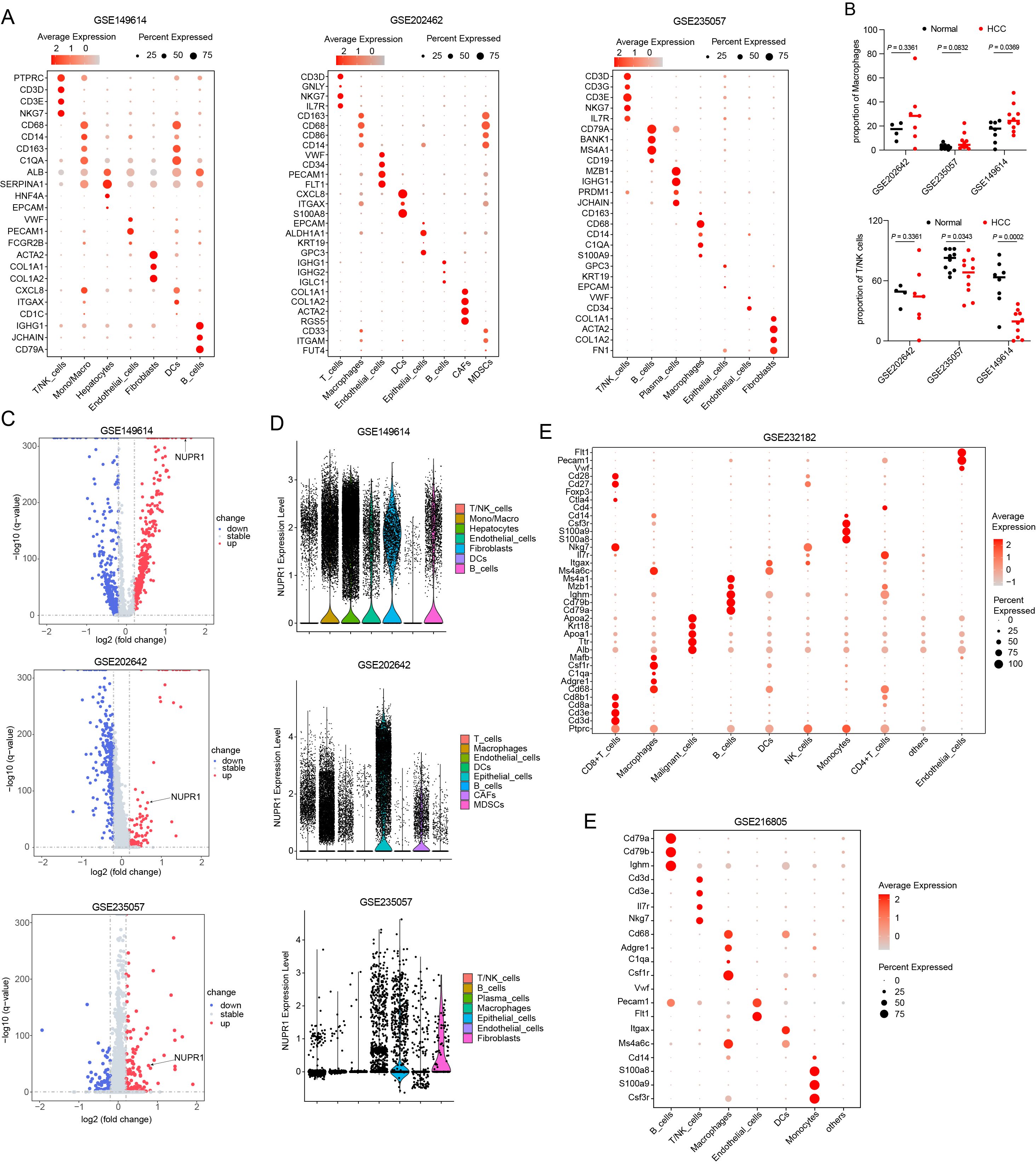


Supplementary Figure1 NUPR1 is highly expressed in TAMs in HCC.

(A): Dot plots showing the specific cell marker for different cell types in human HCC and normal tissue. (B): Bar plots displaying the ratio of macrophage and T/NK cell populations in tumor tissues compared to normal tissues. (C): Volcano Plot of DEGs in macrophages between tumor and normal tissues in three HCC datasets. (D): The violin plot shows NUPR1 expression levels in different cell types. (E): Dot plots showing the specific cell marker for different cell types in murine HCC tumors from GSE232182 dataset. (F) Dot plots showing the specific cell marker for different cell types in murine HCC tumors from GSE216805 dataset. ns, not significant; * *P* < 0.05, ** *P* < 0.01, *** *P* < 0.001.


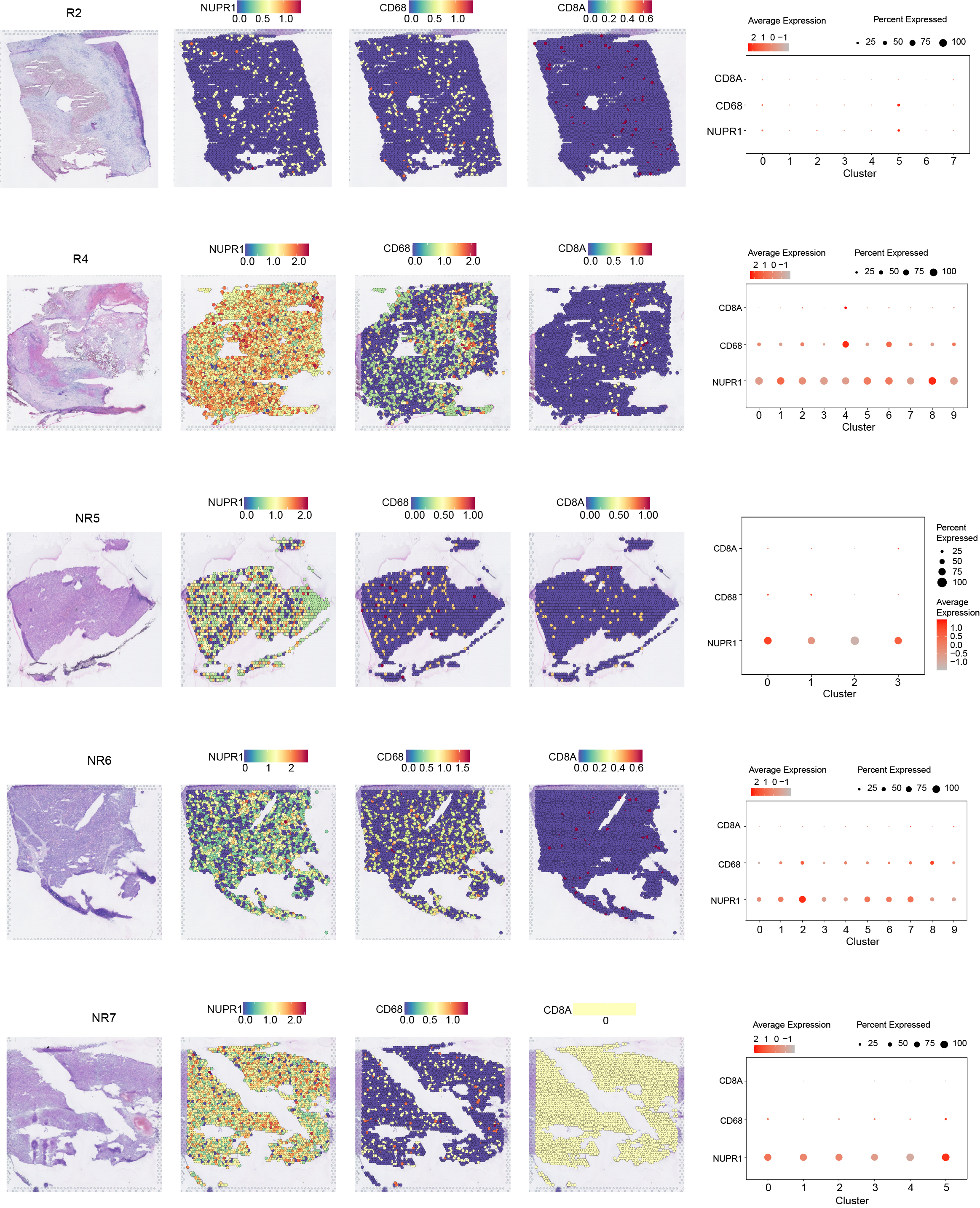


Supplementary Figure2 H-E staining (left) reveals the tissue morphology of spatial transcriptomics samples. The Spatial Violin Plot (middle) shows the expression of NUPR1, CD68, and CD8 across different spatial regions. The Dot Plot (right) illustrates the expression of NUPR1, CD68, and CD8 across various clusters from GSE238264 dataset. ns, not significant; * *P* < 0.05, ** *P* < 0.01, *** *P* < 0.001.


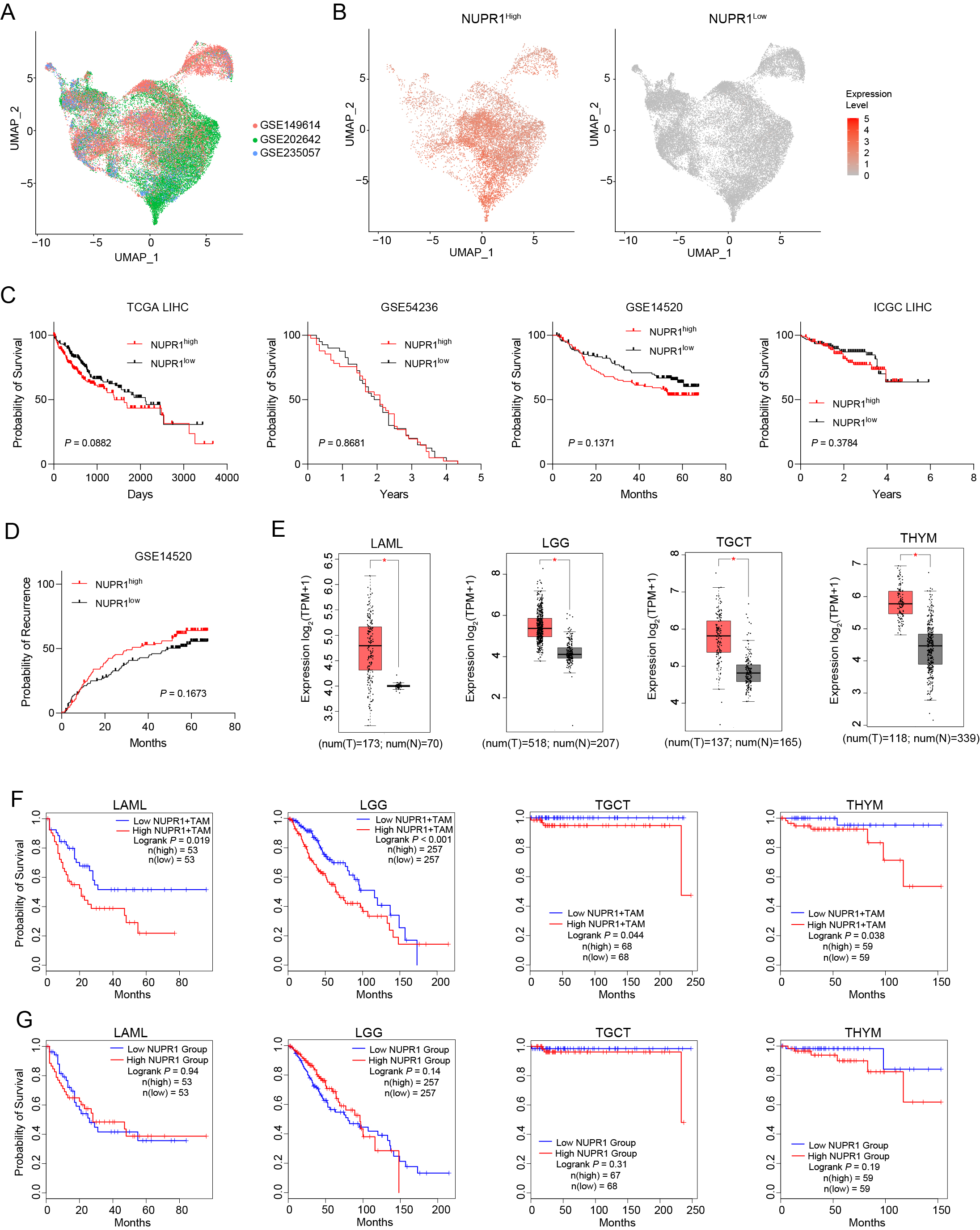


Supplementary Figure3 NUPR1 expression in macrophages mediates poor prognosis in tumors.

(A): UMAP visualization of macrophages, colored by dataset identity. (B): UMAP visualization of macrophages categorized into high and low NUPR1 expression groups based on mean expression value. (C): Kaplan-Meier survival curves illustrating no significant difference in OS between patients with high versus low NUPR1 expression. (D): Kaplan-Meier survival curves illustrating no significant difference in recurrence between patients with high versus low NUPR1 expression. (E): Box plots showing the expression of NUPR1+ macrophages in tumor versus adjacent normal tissues across various cancer types. (F): Kaplan-Meier survival curves comparing the prognosis of patients with high versus low expression of NUPR1+ macrophages across various cancer types. (G): Kaplan-Meier survival curves comparing the prognosis of patients with high versus low NUPR1 expression across various cancer types. ns, not significant; * *P* < 0.05, ** *P* < 0.01, *** *P* < 0.001.


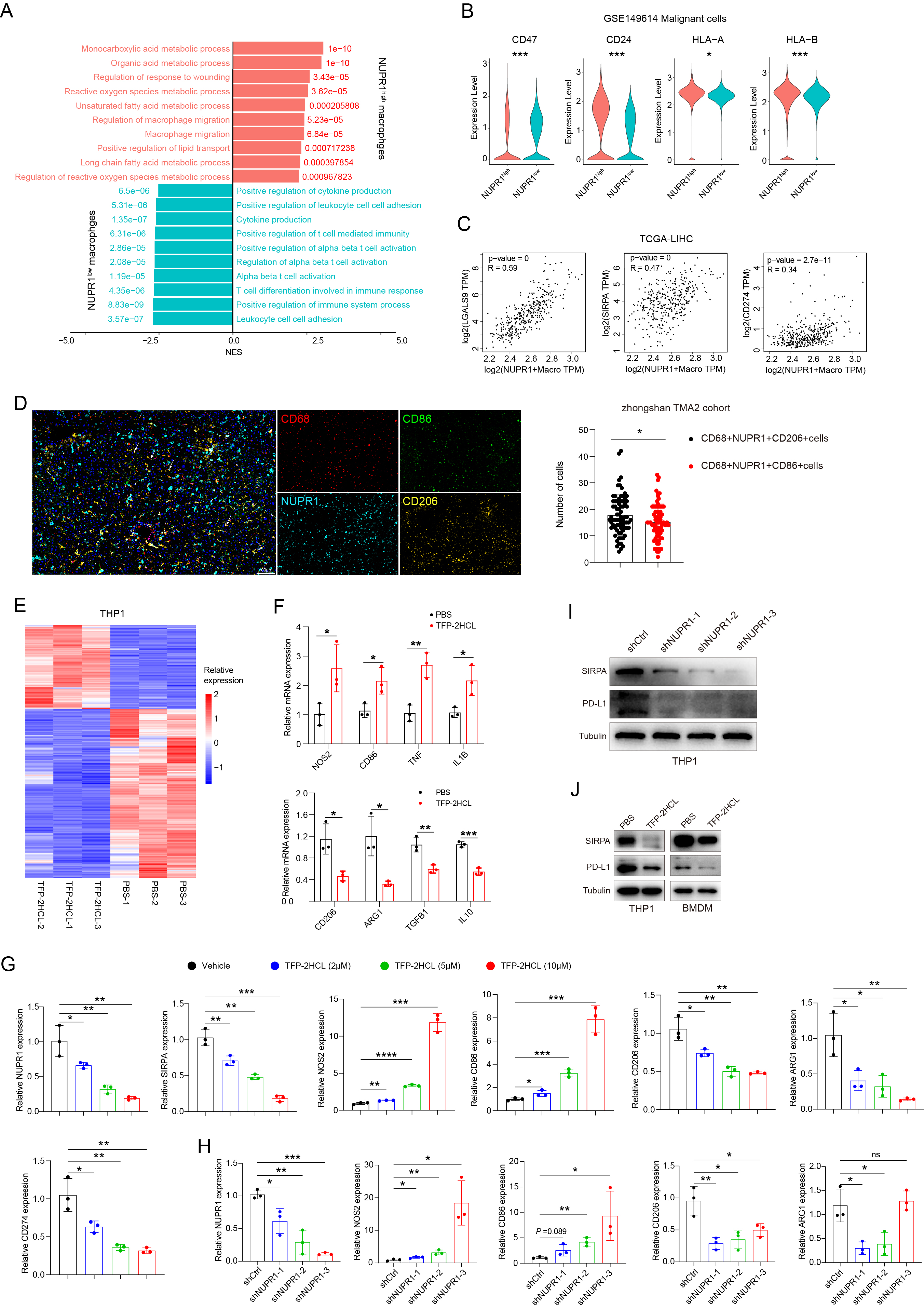


Supplementary Figure4 NUPR1 mediates the immunosuppressive phenotype in macrophages.

(A): GSEA depicting enriched pathways in NUPR1-high macrophages versus NUPR1-low macrophages. (B): Violin plots displaying the expression of 'don't eat me' checkpoint markers in malignant cells from the GSE149614 dataset, categorized by high and low NUPR1 expression levels in macrophages. (C): Correlation analysis showing the relationship between NUPR1+ macrophages and LGALS9, SIRPA, and CD274 in TCGA-LIHC dataset. (D): Representative multiplex immunofluorescence (mIF) staining showing the expression of CD68, CD86, CD206, and NUPR1 in HCC tumor tissues (n = 75). White arrows indicate CD68+CD206+NUPR1+cells, green arrows indicate CD68+CD86+NUPR1+cells, and pink arrows indicate CD68+CD86+CD206+NUPR1+cells. (E): Heatmap displaying differentially expressed genes between THP1 cells treated with TFP-2HCL and control cells. (F): qRT-PCR detected the expression of M1 and M2 macrophage markers in THP1 cells treated with TFP-2HCL (*n* = 3). (G): qRT-PCR detected the expression of M1 and M2 macrophage markers in THP1 cells different concentrations of TFP-2HCL (*n* = 3). (H): qRT-PCR detected the expression of M1 and M2 macrophage markers in THP1 cells transfected with shNUPR1 plasmid or control (*n* = 3). (I): Western blotting showing the expression of SIRPA and CD274 in THP1 cells transfected with shNUPR1 plasmid. (J): Western blotting showing the expression of SIRPA and CD274 in THP1 cells and BMDMs treated with TFP-2HCL. Results are representative of at least three independent experiments. Data are presented as mean ± SD. ns, not significant; * *P* < 0.05, ** *P* < 0.01, *** *P* < 0.001.


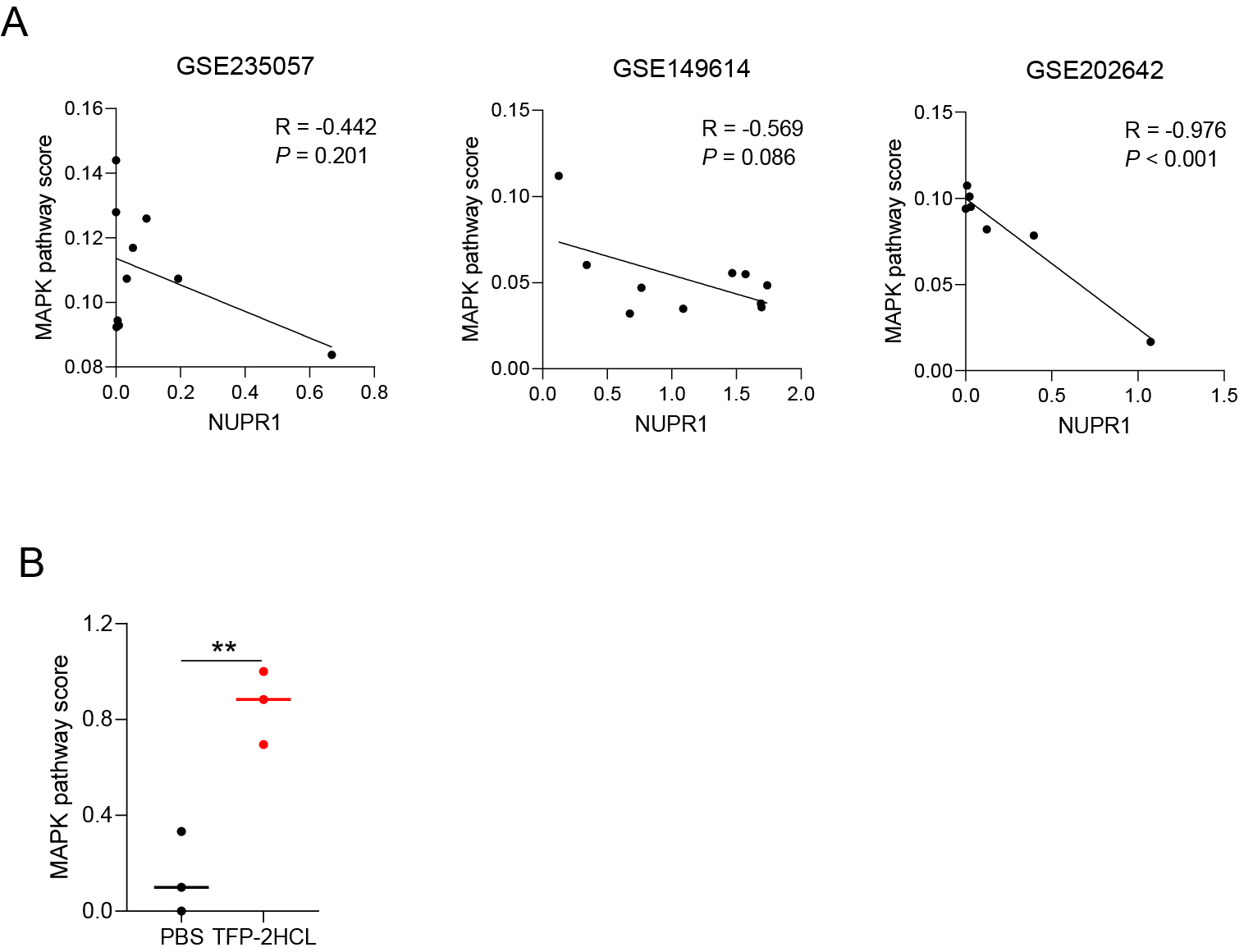


Supplementary Figure5 NUPR1-low macrophages is associated with the MAPK pathways.

(A): Correlation analysis illustrating the association between NUPR1 expression and MAPK pathway scores in macrophages across three scRNA-seq datasets. (B): Bar chart displaying the MAPK pathway scores in TFP-2HCL-treated group compared to the control (*n* = 3). ns, not significant; * *P* < 0.05, ** *P* < 0.01, *** *P* < 0.001.


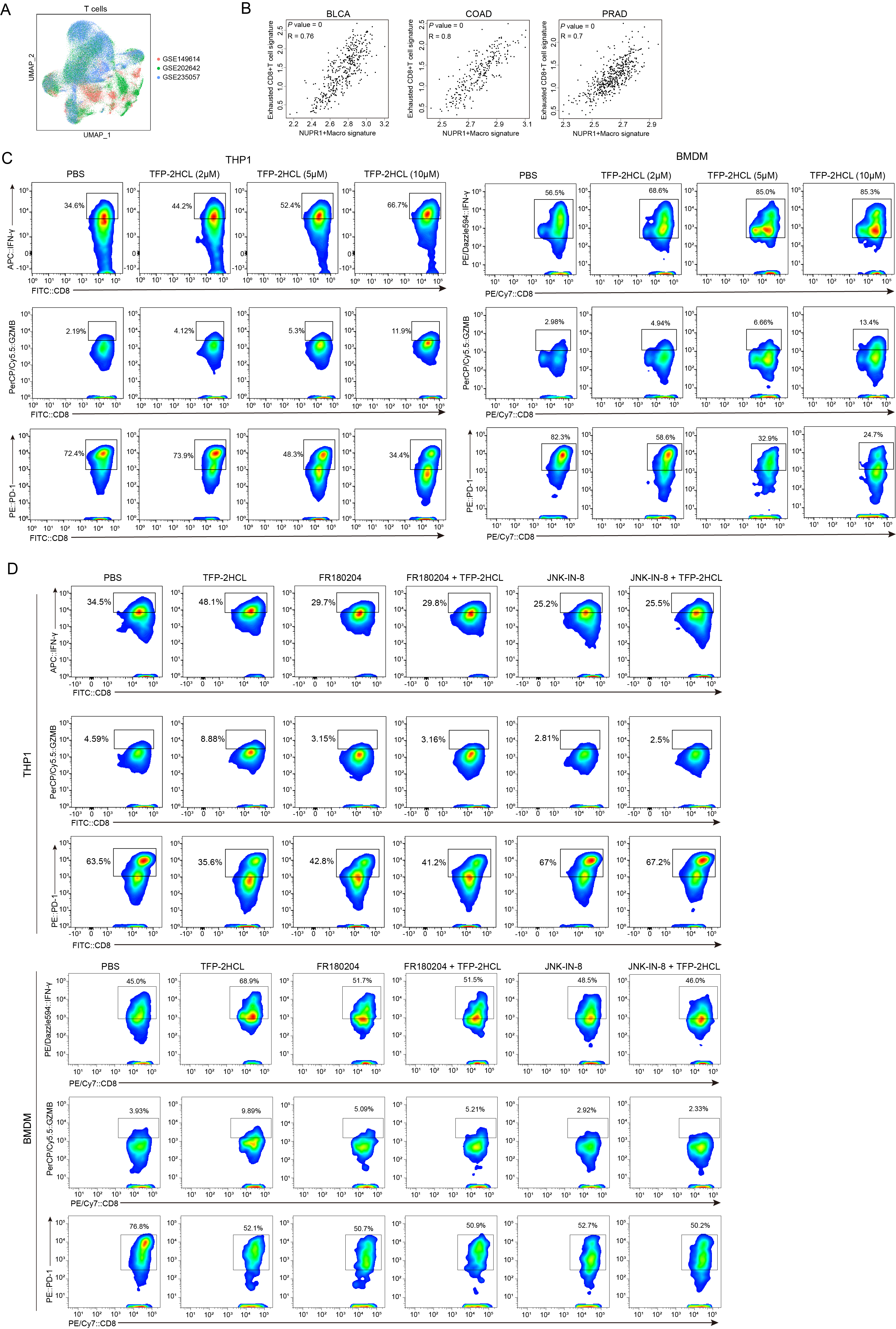


Supplementary Figure6 NUPR1 in macrophages promotes the induction of exhausted CD8+ T cells through the ERK and JNK pathways.

(A): UMAP visualization of CD8+ T cells, colored by dataset identity. (B): Correlation analysis illustrating the association between the expression of NUPR1+ macrophages and exhausted CD8+T cells across different cancer types. (C): Flow cytometric analysis of IFN-γ, Gzmb, and PD-1 on CD8+ T cells co-cultured with macrophages treated with TFP-2HCL (2 µM, 5 µM, and 10 µM) or vehicle. (D) Flow cytometric analysis of IFN-γ, Gzmb, and PD-1 on CD8+ T cells co-cultured with macrophages treated with either TFP-2HCL (10 µM), FR180204 (50 µM), FR180204 (50 µM) + TFP-2HCL (10 µM), JNK-IN-8 (20 µM), JNK-IN-8 (20 µM) + TFP-2HCL (10 µM), or vehicle. ns, not significant; * *P* < 0.05, ** *P* < 0.01, *** *P* < 0.001.


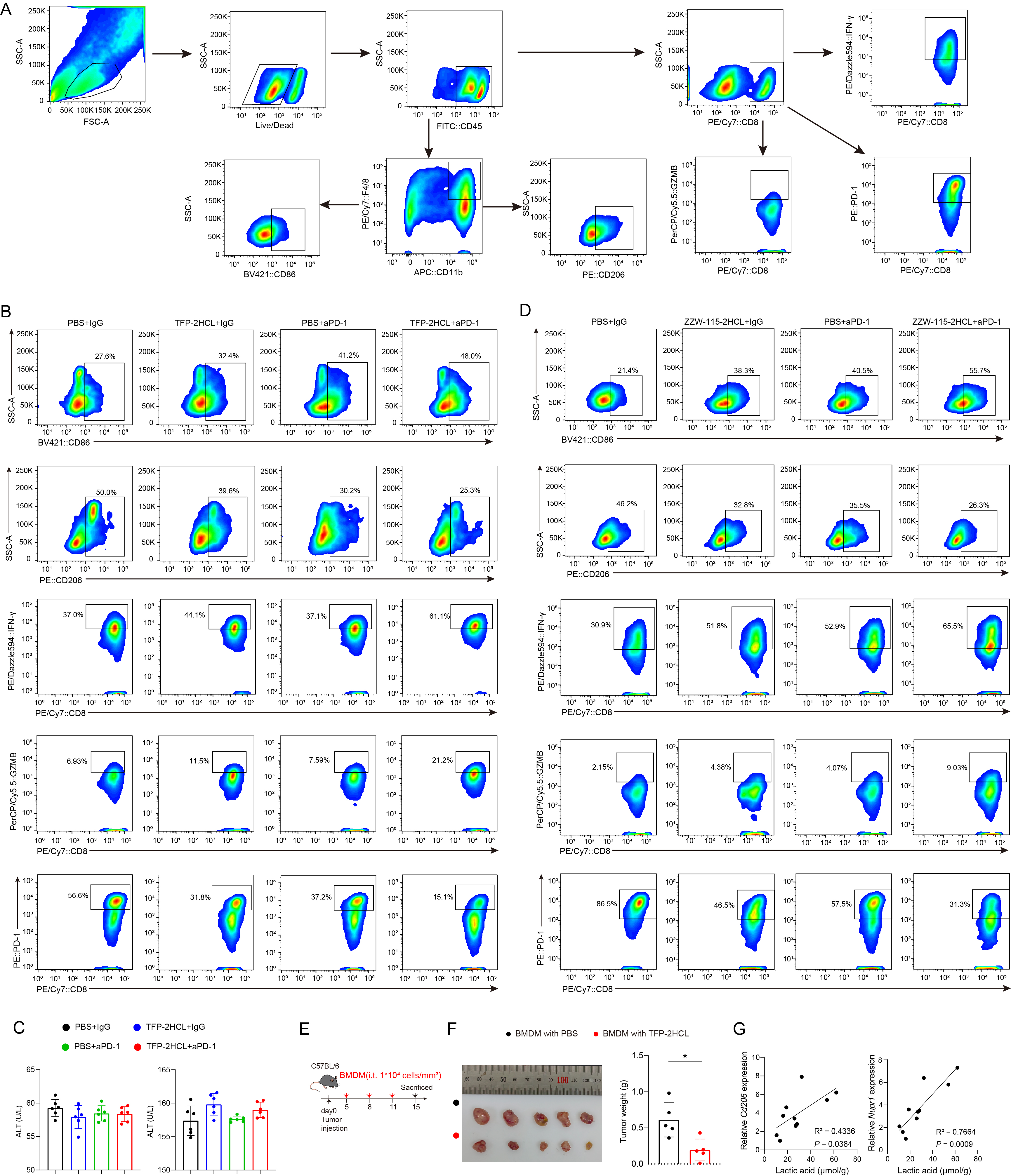


Supplementary Figure7 Targeted inhibition of NUPR1 promotes the development of cytotoxic immune microenvironment.

(A): Gating strategy for various cell populations in murine tumor tissue: CD11b+ F4/80+ macrophages, CD86+ macrophages, CD206+ macrophages, CD45+ CD8+ T cells, IFN-γ+ CD8+ T cells, GZMB+ CD8+ T cells, and PD-1+ CD8+ T cells. (B): Flow cytometric analysis of tumor-infiltrating CD86+ macrophages, CD206+ macrophages, IFN-γ+CD8+ T cells, GZMB+ CD8+ T cells, and PD-1+ CD8+ T cells from subcutaneous HCC tumor (*n* = 6). (C): Quantitative analysis of indicated biochemistry indices for liver function at the endpoint of the experiments (*n* = 6). (D): Flow cytometric analysis of tumor-infiltrating CD86+ macrophages, CD206+ macrophages, IFN-γ+CD8+ T cells, GZMB+ CD8+ T cells, and PD-1+ CD8+ T cells from spontaneous HCC tumor (*n* = 5). (E): Workflow of BMDMs pretreated with PBS or TFP-2HCL for 48h, followed by intratumoral (i.t.) injection as illustrated. (F): Representative tumor images and tumor weight analysis of Hepa1–6 tumors in C57BL/6 mice after i.t. injection of BMDMs pretreated with PBS or TFP-2HCL (*n* = 5 each). (G): Correlation analysis between lactate levels and the expression of CD206 and NUPR1 in tumor tissues. Data are presented as mean ± SD. *n*s, not significant; * *P* < 0.05, ** *P* < 0.01, *** *P* < 0.001.


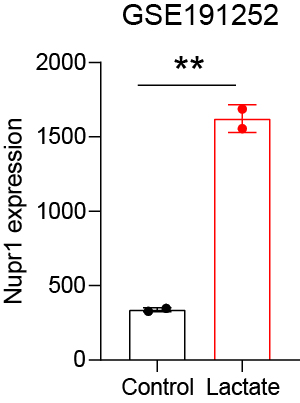


Supplementary Figure8 Lactate is responsible for upregulation of NUPR1 in macrophages. *n*s, not significant; * *P* < 0.05, ** *P* < 0.01, *** *P* < 0.001.


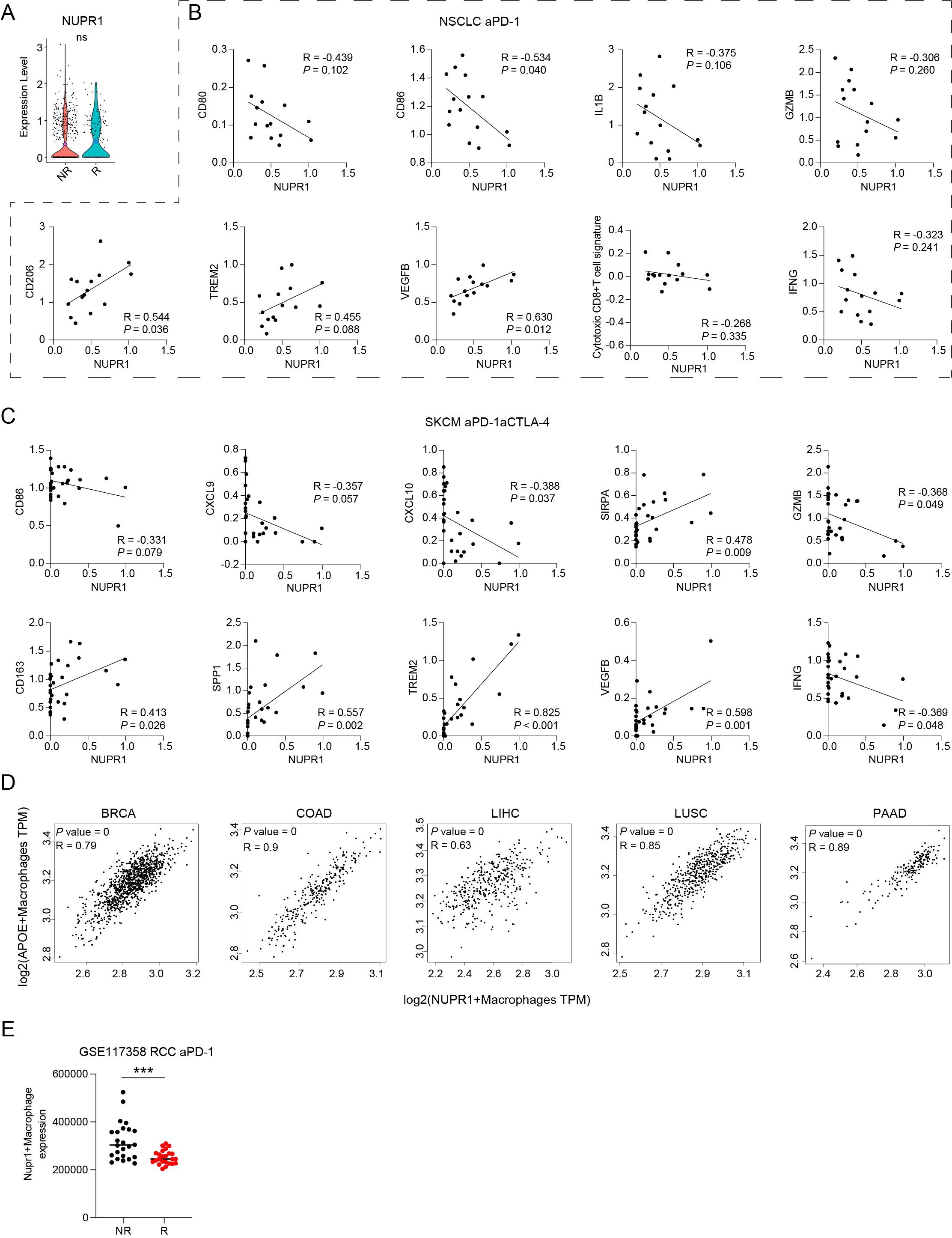


Supplementary Figure9 NUPR1+macrophage is associated with the resistance to immunotherapy.

(A): Violin plots illustrating the expression of NUPR1 in fibroblasts from PD-1 mAb therapy responders and non-responders. (B): Correlation analysis depicting the relationship between NUPR1 expression in macrophages and the expression of CD80, CD86, IL1B, CD206, TREM2, and VEGFB in macrophages, as well as IFN-γ, GZMB, and the cytotoxic CD8+ T cell signature in CD8+ T cells, all within the GSE207422 dataset. (C): Correlation analysis depicting the relationship between NUPR1 expression in macrophages and the expression of M1 and M2 macrophage markers in macrophages, along with IFN-γ and GZMB in CD8+ T cells, all within the GSE120575 dataset. (D): Correlation analysis depicting the relationship between NUPR1+ macrophages and APOE+ macrophages across different cancer types. (E): Bar graph displaying the differential expression of NUPR1+ macrophages between responders and non-responders to immunotherapy in murine renal cell carcinoma datasets. *n*s, not significant; * *P* < 0.05, ** *P* < 0.01, *** *P* < 0.001.

**Supplementary Table S1** Univariate and Multivariate Analyses of Factors Associated with Survival in TCGA-LIHC cohort.

| Variable | Univariate | | | Multivariate | |
| --- | --- | --- | --- | --- | --- |
|  | *P** | HR (95%CI) | *P* | | HR (95%CI) |

| Age, year (≥60 vs. <60) | **0.042** | 1.617(1.018-2.567) | | **0.031** | | 1.708(1.049-2.81) | |
| --- | --- | --- | --- | --- | --- | --- | --- |
| Gender (Female vs. Male) | **0.047** | 0.631(0.401-0.993) | | 0.482 | | 0.841(0.518-1.364) | |
| AFP (≥20 vs. <20, ug/L) | 0.076 | 1.506(0.958-2.367) | | 0.656 | | 1.118(0.685-1.823) | |
| Grade (I vs. II vs. III) | **0.019** | 1.450(1.063-1.979) | | **0.019** | | 1.509(1.071-2.126) | |
| AJCC stage | **0.005** | 1.427(1.112-1.830) | | 0.872 | | 1.081(0.518-1.364) | |
| Child Pugh grade (A vs. B&C) | 0.369 | 1.430(0.655-3.120) | | 0.142 | | 1.866(0.82-4.289) | |
| NUPR1+Macrophage | **0.007** | 1.886(1.188-2.995) | **0.023** | | 1.746(1.079-2.827) | |  |

Abbreviations: HR, hazard ratio. *, *P* < 0.05 was regarded as statistically significant, *P* value was calculated using Cox’s proportional hazards regression

**Supplementary Table S2.** Sequences of the primers used in the study

| Gene | | | Sequences (5'- -3') |
| --- | --- | --- | --- |
| qPCR | | |  |
| NUPR1 | Forward | CTCTCATCATGCCTATGCCTACT | |
|  | Reverse | CCTCCACCTCCTGTAACCAAG | |
| ACTB | Forward | GATCATTGCTCCTCCTGAGC | |
|  | Reverse | ACTCCTGCTTGCTGATCCAC | |
| CD86 | Forward | CTGCTCATCTATACACGGTTACC | |
|  | Reverse | GGAAACGTCGTACAGTTCTGTG | |
| IL1B | Forward | ATGATGGCTTATTACAGTGGCAA | |
|  | Reverse | GTCGGAGATTCGTAGCTGGA | |
| TNF | Forward | CCTCTCTCTAATCAGCCCTCTG | |
|  | Reverse | GAGGACCTGGGAGTAGATGAG | |
| NOS2 | Forward | AGGGACAAGCCTACCCCTC | |
|  | Reverse | CTCATCTCCCGTCAGTTGGT | |
| CD206 | Forward | GGGTTGCTATCACTCTCTATGC | |
|  | Reverse | TTTCTTGTCTGTTGCCGTAGTT | |
| ARG1 | Forward | TGGACAGACTAGGAATTGGCA | |
|  | Reverse | CCAGTCCGTCAACATCAAAACT | |
| IL10 | Forward | TCAAGGCGCATGTGAACTCC | |
|  | Reverse | GATGTCAAACTCACTCATGGCT | |
| TGFB1 | Forward | CTAATGGTGGAAACCCACAACG | |
|  | Reverse | TATCGCCAGGAATTGTTGCTG | |
| Nupr1 | Forward | CCCTTCCCAGCAACCTCTAAA | |
|  | Reverse | TCTTGGTCCGACCTTTCCGA | |
| Cd206 | Forward | CTCTGTTCAGCTATTGGACGC | |
|  | Reverse | CGGAATTTCTGGGATTCAGCTTC | |
| Actb | Forward | TGACGTTGACATCCGTAAAGA | |
|  | Reverse | GCCGGACTCATCGTACTCC | |
| ChIP-qPCR |  |  | |
| NUPR1 | Forward | GTCCCACATTCCACAGAGGG | |
|  | Reverse | TCTCTTGGTGCGACCTTTCC | |
| Nupr1 | Forward | AAGCAGGACAACCTGTGCTT | |
|  | Reverse | ACACAGCTTGCAGTTGGTTC | |

## Supplementary Table S3 Antibodies used in this study

| Antibody | Dilution ratio | Cat# | Supplier |
| --- | --- | --- | --- |
| NUPR1 | 1:300 for WB | 15056-1-AP | Proteintech |
| Tubulin | 1:3000 for WB | MA5-16308 | Thermo Fisher Scientific |
| Anti-p38 MAPK | 1:1000 for WB | T55600 | Abmart |
| Anti-Phospho-p38 MAPK | 1:1000 for WB | TP56391 | Abmart |
| Anti-ERK1/2 | 1:1000 for WB | T40071 | Abmart |
| Anti- Phospho-Erk1(T202/Y204) +Erk2(T185/Y187) | 1:1000 for WB | T40072 | Abmart |
| Anti-JNK1/2/3 | 1:1000 for WB | T40073 | Abmart |
| Anti- p-JNK1/2/3 (Thr183+Tyr185) | 1:1000 for WB | T40074 | Abmart |
| anti-Histone H3 | 1:1000 for WB | ab176842 | Abcam |
| anti-L-Lactyl Lysine | 1:1000 for WB | PTM-1401RM | PTM BIO |
| anti-Lactyl-Histone H3(Lys18) | 1:1000 for WB | PTM-1406RM | PTM BIO |
| anti-Lactyl-Histone H3(Lys18) | 6μL per IP | PTM-1427RM | PTM BIO |
| CD16/CD32 Monoclonal Antibody | 1:50 for FC | MFCR00-4 | Thermo Fisher Scientific |
| PE-Cy7 anti-Rabbit F4/80 | 1:100 for FC | 25-4801-82 | Thermo Fisher Scientific |
| PE anti-Rabbit CD206 | 1:400 for FC | 12-2061-80 | Thermo Fisher Scientific |
| APC anti-Rabbit CD11b | 1:400 for FC | 17-0112-82 | Thermo Fisher Scientific |
| BV421 anti-mouse CD86 | 1:40 for FC | 105032 | BioLegend |
| FITC anti-human CD86 | 1:40 for FC | 374204 | BioLegend |
| FITC anti-Mouse CD45 | 1:400 for FC | 103108 | BioLegend |
| PE-Cy7 anti-Mouse CD8 | 1:200 for FC | 100722 | BioLegend |
| FITC anti-Human CD8 | 1:200 for FC | 980908 | BioLegend |
| PE-Dazzle594 anti-Mouse IFN-γ | 1:200 for FC | 505846 | BioLegend |
| APC anti-Human IFN-γ | 1:200 for FC | 986702 | BioLegend |
| PerCP-Cy5.5 anti- Human /Mouse Gzmb | 1:40 for FC | 372211 | BioLegend |
| PE anti-Mouse PD-1 | 1:200 for FC | 135205 | BioLegend |
| PE anti-Human PD-1 | 1:200 for FC | 379209 | BioLegend |
| PE anti-Human CD206 | 1:40 for FC | 321105 | BioLegend |
| eBioscience™ Fixable Viability Dye eFluor™ 506 | 1:200 for FC | 65-0866-14 | Thermo Fisher Scientific |
| InVivoMab anti-Mouse PD-1 | 200 μg per time | BE0146 | Bio X Cell |
| InVivoMab rat IgG2a isotype control | 200 μg per time | BE0089 | Bio X Cell |
| HRP anti-Mouse IgG | 1:10000 for WB | RS0001 | Immunoway |
| HRP anti-Rabbit IgG | 1:10000 for WB | RS0002 | Immunoway |

Abbreviations: WB, western blotting; IP, immunoprecipitation; FC, flow cytometry.

**Supplementary Table S4** Sequences for shRNA used in this study

| **Sequences for shRNA** | (5’-3’) |
| --- | --- |
| shCtrl | CCTAAGGTTAAGTCGCCCTCG |
| shNUPR1-1 | AAGAAAGCTACAGAAGAAACT |
| shNUPR1-2 | GAGGAAACTGGTGACCAAGCT |
| shNUPR1-3 | GGATGAATCTGACCTCTATAG |
